# A GC-IRMS method for measuring sulfur isotope ratios of carbonyl sulfide from small air samples

**DOI:** 10.12688/openreseurope.13875.2

**Published:** 2022-03-08

**Authors:** Sophie L. Baartman, Maarten C. Krol, Thomas Röckmann, Shohei Hattori, Kazuki Kamezaki, Naohiro Yoshida, Maria Elena Popa

**Affiliations:** 1 Institute for Marine and Atmospheric research Utrecht (IMAU), Utrecht University, Utrecht, 3584 CS, The Netherlands; 2 Meteorology and Air Quality, Wageningen University & Research Center, Wageningen, 6708 PB, The Netherlands; 3 Department of Chemical Science and Engineering, School of Materials and Chemical Technology, Tokyo Institute of Technology, Yokohama, 226-8502, Japan; 4 International Center for Isotope Effects Research (ICIER), Nanjing University, Nanjing, 210023, China; 5 Department of Material and Life Sciences, Faculty of Science & Technology, Sophia University, Tokyo, 102‐8554, Japan; 6 Environmental Management Research Institute, National Institute of Advanced Industrial Science and Technology (AIST), Tsukuba, 305-8569, Japan; 7 Earth-Life Science Institute, Tokyo Institute of Technology, Tokyo, 152-8550, Japan; 8 National Institute of Information and Communications Technology, Tokyo, 184-8795, Japan

**Keywords:** carbonyl sulfide, sulfur isotope, atmosphere, biosphere, mass spectrometry

## Abstract

A new system was developed for measuring sulfur isotopes δ ^33^S and δ ^34^S from atmospheric carbonyl sulfide (COS) on small air samples of several liters, using pre-concentration and gas chromatography – isotope ratio mass spectrometry (GC-IRMS). Measurements of COS isotopes provide a tool for quantifying the COS budget, which will help towards better understanding climate feedback mechanisms. For a 4 liter sample at ambient COS mixing ratio, ~500 parts per trillion (ppt), we obtain a reproducibility error of 2.1 ‰ for δ ^33^S and 0.4 ‰ for δ ^34^S. After applying corrections, the uncertainty for an individual ambient air sample measurement is 2.5 ‰ for δ ^33^S and 0.9 ‰ for δ ^34^S. The ability to measure small samples allows application to a global-scale sampling program with limited logistical effort. To illustrate the application of this newly developed system, we present a timeseries of ambient air measurements, during the fall and winter of 2020 and 2021 in Utrecht, the Netherlands. The observed background values were δ ^33^S = 1.0 ± 3.4 ‰ and δ ^34^S = 15.5 ± 0.8 ‰ (VCDT). The maximum observed COS mixing ratios was only 620 ppt. This, in combination with the relatively high δ ^34^S suggests that the Netherlands receives little COS-containing anthropogenic emissions. We observed a change in COS mixing ratio and δ ^34^S with different air mass origin, as modelled with HYSPLIT backward trajectory analyses. An increase of 40 ppt in mean COS mixing ratio was observed between fall and winter, which is consistent with the expected seasonal cycle in the Netherlands. Additionally, we present the results of samples from a highway tunnel to characterize vehicle COS emissions and isotopic composition. The vehicle emissions were small, with COS/CO _2_ being 0.4 ppt/ppm; the isotopic signatures are depleted relatively to background atmospheric COS.

## Introduction

Carbonyl sulfide (COS) is the most abundant sulfur-containing trace gas in the atmosphere, with an average mixing ratio of 500 parts per trillion (ppt) ( Chin & Davis, 1995). It has a lifetime of around two years, which permits it to be transported into the stratosphere. There, it is likely the main source of stratospheric sulfur aerosols (SSA) during volcanically quiescent periods. These aerosols regulate the Earth’s albedo and play a crucial role in stratospheric chemistry ( Brühl *et al*., 2012; Crutzen, 1976; Kremser *et al*., 2016). Understanding the role of COS in stratospheric chemistry is therefore highly important for understanding cooling mechanisms of the Earth.

Another way in which COS can be used to better understand the climate system is through its potential use for the quantification of gross primary production (GPP) of the biosphere. It is difficult to derive GPP from measurements of CO _2_, because flux measurements only yield the sum the two almost cancelling fluxes: GPP and respiration. COS, however, is taken up by plants in an essentially one-way reaction, during which it follows almost the same pathway as CO _2_ ( Protoschill-Krebs & Kesselmeier, 1992; Protoschill-Krebs *et al*., 1996; Whelan *et al*., 2018). Therefore, measurements of the unidirectional COS uptake could be used as a tracer for photosynthetic CO _2_ uptake and help to quantify GPP ( Asaf *et al*., 2013; Berry *et al*., 2013; Blonquist *et al*., 2011; Campbell *et al*., 2008; Kooijmans *et al*., 2016).

The largest natural source of COS is the ocean, in the form of direct emission and indirect emission via carbon disulfide (CS _2_) and possibly dimethyl sulfide (DMS) ( Kettle *et al*., 2002; Lennartz *et al*., 2017; Lennartz *et al*., 2020). The other main sources of COS are anthropogenic, and include rayon production, aluminum production, coal combustion and other smaller sources such as biomass burning ( Stinecipher *et al*., 2019; Zumkehr *et al*., 2018). Sinks of COS include the above-mentioned large biosphere uptake, and a smaller and less well characterized soil uptake sink ( Whelan *et al*., 2018). Unfortunately, the budget of COS is still not well understood and large uncertainties exist in the strengths of the sinks and particularly the sources of COS ( Whelan *et al*., 2018). Modelling studies and satellite observations can help further constrain the COS budget ( Barkley *et al*., 2008; Glatthor *et al*., 2017; Kettle *et al*., 2002; Kuai *et al*., 2014; Suntharalingam *et al*., 2008; Yousefi *et al*., 2019) but the latest studies still point to an unknown missing source of 230 – 432 Gg S a ^-1^ ( Ma *et al*., 2021). Isotopic measurements could provide a tool for overcoming these budget uncertainties, as they can be used to characterize source and sink contributions. Different types of COS sources have distinct sulfur isotopic compositions, which can be used to identify these sources. In COS removal reactions, the lighter isotope is usually preferred over the heavier one because of differences in chemical bond strength. These principles can be used to characterize the influences of sources and sinks. The sulfur isotope ratios are reported as δ values, which are defined by Equation 1 and Equation 2, where R is the ratio between the heavier and the lighter isotope, which is then used to calculate the δ ^33^ and δ ^34^ values. The δ values are reported relative to the international sulfur standard; the Vienna Canyon Diablo Troilite (VCDT).

$${\;}^{33,34}R=[{\;}^{33,34}S]/[{\;}^{32}S]\;(1)$$

$$\delta {\;}^{33,34}S=\frac{R_{\text{sample}}^{33,34}}{R_{\mathrm{standard}}^{33,34}}-1\;(2)$$

To date, two methods have been developed for measuring the sulfur isotopic composition of COS. The first method, described by Hattori *et al*. (2015) and Kamezaki *et al*. (2019), uses gas chromatography - continuous flow isotope ratio mass spectrometry (GC-CF-IRMS) to measure S ^+^ fragment ions, and requires very large air samples of several hundreds of liters. This is because of the dependence of the isotope values on the sample amount, usually referred to as non-linearity, that arises when using smaller sample sizes. The second method was first presented by Angert *et al*. (2019) and uses a multi collector inductively coupled plasma mass spectrometer (MC-ICP-MS), which can measure sulfur isotopic composition of COS from smaller sample sizes of around 3 L. The method we present here is in principle similar to the CF-IRMS method of Hattori *et al*. (2015), but optimized for small sample volumes of 3 – 4 L of air. This is possible because we characterized the nonlinearity of our system and we apply a correction factor to our isotope measurements that accounts for this nonlinearity.

Newman *et al*. (1991) presented the first global estimate of δ ^34^S in tropospheric COS of 11 ‰, based on mass-balance calculations. Angert *et al*. (2019) measured COS sulfur isotopologues from clean ambient air at the Canary Islands and Israel. They found a mean δ ^34^S of 13.4 ± 0.5‰, which is roughly in agreement with the estimate by Newman *et al*., 1991. Kamezaki *et al*. (2019) showed results from four measurements in Yokohama, with a mean δ ^34^S of 10.5 ± 0.4 ‰. They explained their slightly lower δ ^34^S than Angert *et al*. (2019) by the presence of anthropogenic COS emissions from China, which have an estimated lower δ ^34^S value of 3 to 8 ‰ ( Davidson *et al*., 2021; Hattori *et al*., 2020). In a later paper, Hattori *et al*. (2020) presented new results from air collected at three different locations at different latitudes in Japan during both winter and summer. They found significantly higher mixing ratios and lower δ ^34^S values for the most northerly location in winter, which predominantly received air from highly industrialized regions in China. In addition, they found higher δ ^34^S values when the air was predominantly coming the East, where the ocean source dominates. Based on these results and the Keeling plot intercepts ( Keeling, 1961; Pataki *et al*., 2003), Hattori *et al*. (2020) deduced an anthropogenic emission value for δ ^34^S of 4 to 5 ‰ and a value of 19 ‰ for the ocean source. These results are roughly in agreement with the newest results presented by Davidson *et al*. (2021), who measured COS mixing ratios and δ ^34^S from 89 air samples from multiple locations around the world. By dividing their dataset in high (>600 ppt) and low (<600 ppt) mixing ratio data and calculating the Keeling plot intercepts they found an anthropogenic source value of 8.1 ± 1 ‰. Davidson *et al*. (2021) also measured direct and indirect COS emissions from the Mediterranean Sea and found a combined δ ^34^S signature of 13.2 ± 2 ‰ for the ocean source. Thus, the ocean source δ ^34^S signature is estimated to be between 13 and 19 ‰ ( Davidson *et al*., 2021; Hattori *et al*., 2020). The anthropogenic emission signature is estimated to be slightly lower than the ambient δ ^34^S and ranges between 4 and 8 ‰. The biosphere fractionation sink was estimated by Davidson *et al*. (2021) in a plant chamber experiment, and yielded a fractionation factor ^34^ε of –1.9 ± 0.3 ‰ for one plant species, thus making the remaining COS pool enriched in δ ^34^S. The fractionation during uptake by three different types of soil bacteria has been measured by Kamezaki *et al*. (2016) and Ogawa *et al*. (2017) and they found a similar small negative ^34^ε between –3.7 and –2.1 ‰. The destruction reaction of COS with hydroxyl radicals (OH) will likely make the COS more enriched in the heavier sulfur and carbon isotopes ( Schmidt *et al*., 2012). An experimental study by Hattori *et al*. (2011) found sulfur isotopic fractionation factors for photolysis destruction of COS of – 3.7 ± 4.5 ‰ and 1.1 ± 4.2 ‰ for ^33^ε and ^34^ε, respectively. The sulfur isotope effects during the reaction with atomic oxygen (O( ^3^P)) were investigated by Hattori *et al*. (2012) and a fractionation factor ^34^ε = –21.7 ± 6.2 ‰ was found. However, large uncertainties in these values remain and more research is needed on the isotopic compositions or fractionation factors of sources and sink processes of COS, such as traffic and biomass burning emissions, destruction by atmospheric oxidation and photolysis in the stratosphere.

In summary, substantial progress has been made in the last years on measuring COS sulfur isotopologues. However, in order to fully characterize the global COS budget and its sulfur isotopic composition, more measurements are needed, including atmospheric measurements from several different climatic zones, latitudes and altitudes ( Ma *et al*., 2021). This paper presents the methodology and first results of our new system that can measure sulfur isotopes of COS using CF-IRMS at Utrecht University, the Netherlands. We present results from online ambient air measurements at the Utrecht University campus, over a time-span of five months in the fall and winter of 2020/2021. We also provide an estimate on the isotopic signature of COS vehicle emissions from air samples taken in a highway tunnel.

## Methods

### Measurement system

**Figure 1.**
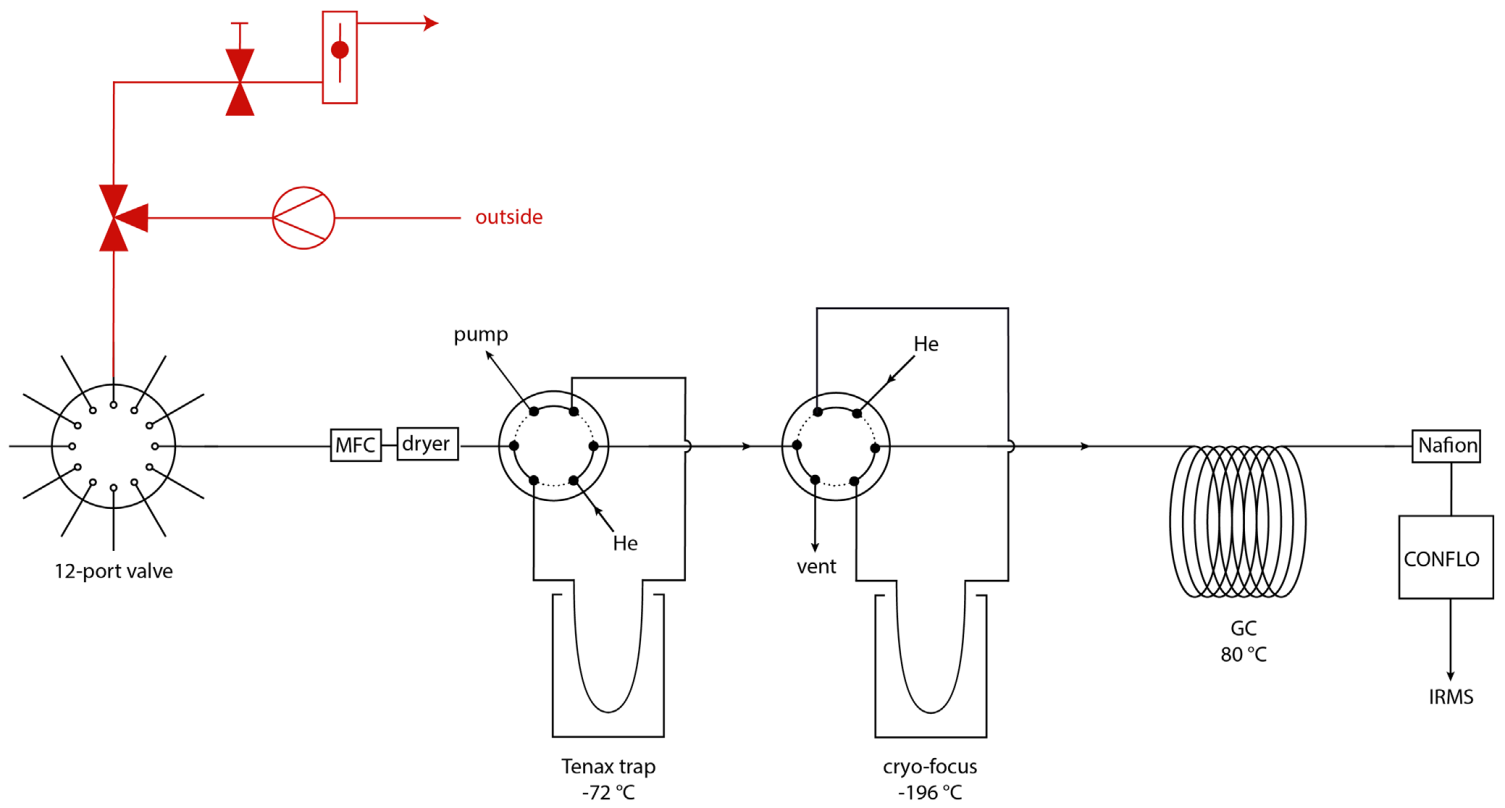
Schematic overview of the COS pre-concentration system, with in red the ambient air sampling system including a three-way valve, a pump, a needle valve and a flow controller and in black the pre-concentration system, with a 12-port dead-end multi-position valve, a mass flow controller (MFC), a magnesium perchlorate dryer, two 6 port valves, a Tenax TA trap and a cryo-focus trap, a gas chromatographic column (GC), a Nafion dryer, a ConFlo interface and an isotope ratio mass spectrometer.

Figure 1 shows a schematic overview of the pre-concentration coupled with a CF-IRMS system, that was developed at the Institute for Marine and Atmospheric research Utrecht (IMAU). The system is partially similar to the ones described in Hattori *et al*. (2015), Kamezaki *et al*. (2019) and Angert *et al*. (2019) and consists of several traps to collect the COS, while discarding the other air compounds. In short, the sample gas is first directed through a cooled Tenax trap, where the COS is preferentially collected. The collected gas is then transferred to the cryo-focus and afterwards further purified in a GC column, before being sent via an open split system to the IRMS for isotope ratio measurements.

Samples, reference gases and COS-free “zero” air for blank measurements are connected to the system with a 12 port multi-position selection valve (EMT2CSD12MWE, dead-end path, 200C/400 psi, Vico Valco Instruments Co. Inc., USA). The selected sample is passed through a dryer, which consists of a glass tube containing magnesium perchlorate (Mg(ClO _2_) _4_) (63095, Fluka Analytical, Switzerland), held in place by silane treated glass wool at both ends of the tube. As we mostly measure already pre-dried samples, the dryer is replaced approximately every three to four months, when the material starts to look moist. The Tenax trap consists of a 1/16” sulfinert-treated tube (29229, Restek, USA) filled with approximately 200 mg of Tenax TA (60 – 80 mesh, 11982, Supelco Analytical, USA) and silane treated glass wool (20411, Supelco, USA), and is cooled with a mixture of ethanol and dry ice to –72 °C to trap COS. The Tenax trap also contains some 1mm diameter glass beads to allow for slightly larger pore spaces, to reduce the flow resistance of the trap. Before use, the Tenax is conditioned for 24 hours at a temperature of 200 °C under helium flow. After every measurement, the Tenax trap is flushed forward with a helium flow of 30 mL/min and heated to 200 °C for 30 minutes, in order to limit memory from the previous sample. Sample flow into the trap is kept at or below 80 mL/min using a mass flow controller, depending on the pressure of the sample being measured. A membrane vacuum pump (type N920 G, KNF, France) is used at the outlet of the trap in order to maintain a high enough flow rate, even when measuring samples with lower than atmospheric pressure. After the desired volume of sample gas is injected in the Tenax trap, the collected gas is released from the Tenax by heating the trap to 130 °C with a heating wire. The gas is transported with helium carrier gas, through a six-port valve (A4C6UWM, Vici Valco Instruments Co. Inc., USA) to the cryo-focus trap, where the gas is collected for 30 min at a temperature of –196 °C, using liquid nitrogen. This second cold trap consists of a 320 *μ*m inner diameter capillary tube (P/N 064052, Trajan, Australia) and is used to focus the COS in a smaller volume and release it all at the same time, creating a narrower peak on the chromatograph than if only the first cold trap would be used, which is larger and heats up slower. After this focusing step, the remaining compounds in the gas mixture are separated on a gas chromatographic column (CP7549, PoraPLOT Q, 25m, 0.25mm, Agilent Technologies, USA) heated to 80 °C. A ConFlo IV universal continuous flow interface (IQLAAEGAATFAETMAXB, Thermo Fisher Scientific, USA) is used to inject the gas into the IRMS (IQLAAEGAATFABHMZZZ, Delta V Advantage, Thermo Fisher Scientific, USA), where the COS is ionized and fragmented, with a S ^+^ fragment yield of approximately 30 % ( NIST, 2018). The fragment ions ^32^S ^+^, ^33^S ^+^ and ^34^S ^+^ are collected on triple Faraday collector cups for m/z 32, 33 and 34, with resistors 3 * 10 ^9^ Ω, 1 * 10 ^12^ Ω and 3 * 10 ^11^ Ω. Altogether, one sample measurement takes 2 to 3 hours, depending on initial pressure and the volume of sample that was injected. The injected sample volume was chosen based on the expected COS mixing ratio in the sample, and adjusted so that the sample COS peak area would be similar to the reference gas COS peak area.

 At the start of each measurement, a working gas is injected into the IRMS via the ConFlo three times. As working gas, pure O _2_ is used, which has the three isotope masses needed. Additionally, since pure COS is highly toxic, it is much safer and more convenient to use O _2_ as a working gas. The isotope ratios of all sample and reference measurements are first calculated relative to our working gas. From these, the sample values are calculated relative to the reference gas, which is calibrated against the international standard VCDT ( NIST, 2013). This calibration process and other data corrections and processing are further elaborated in the next section.

**Figure 2.**
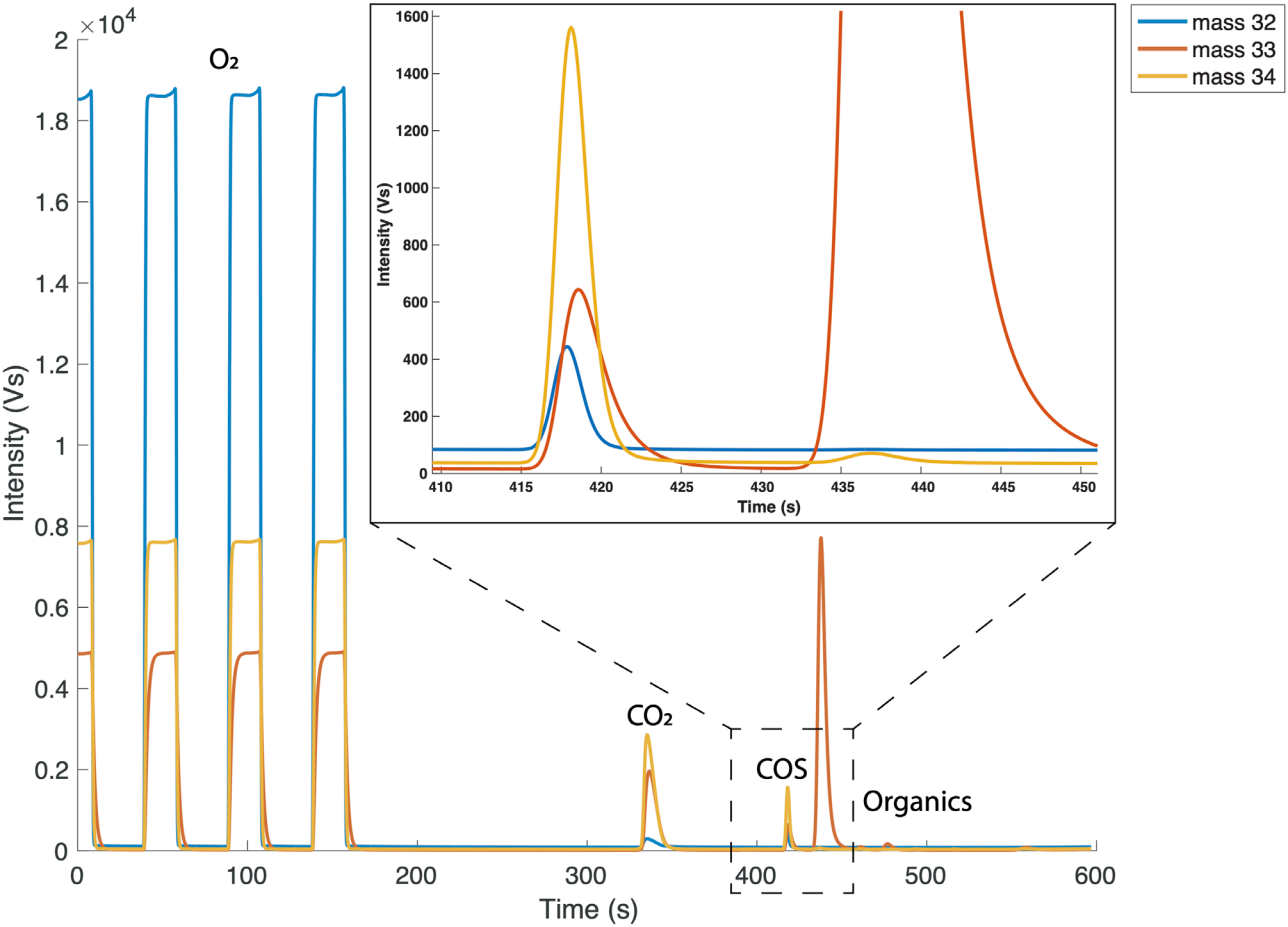
Full chromatograph of one reference gas measurement. First three peaks are the O _2_ working gas peaks. The chromatogram starts with O _2_ in the source flushing because the IRMS source is constantly flushed with O _2_ in between measurements. After the working gas peaks we see a peak which is suspected to be CO _2_, which is followed by the small COS peak, and several other peaks that appear on m/z 33 only. The inset in the top right corner zooms in on the COS peak.

Figure 2 shows an example of a chromatogram of a COS measurement with the three square peaks of the working gas at the beginning, followed by additional peaks including the COS sample peak. The peak that arrives after the O _2_ square peaks, with a retention time of around 320 seconds, is likely from the O _2_^+^ fragment of remaining CO _2 _that is also being trapped in the Tenax trap, as this peak increases for gases with elevated CO _2_ concentrations. Most of the CO _2_ that is trapped on the Tenax is removed through timed valve switching, thus we expect that the peak on the chromatogram is only the “tail” of the peak. At 410 seconds, the COS peak elutes, with the m/z of 33 and 34 traces having larger amplitudes than *m*/ *z* 32 trace because of the much higher resistors. The several peaks that follow the COS peak, but are well separated, mostly show on m/z 33 and are possibly organic compounds as mentioned by Kamezaki *et al*. (2019).

### Testing the pre-concentration system

***Reproducibility***. The reproducibility of the system was characterized by measuring 1 L, 2 L and 3 L volumes of the same gas with 6 to 8 repetitions, using the same method for all measurements. The gases used were target gas 2 (ambient air reference gas for CO isotope measurements) and a 5 L stainless steel cylinder filled with ambient air during EastGrip 2018 in Greenland. From these measurements, we estimated the typical reproducibility as the two-tailed 70% confidence interval using the student t-distribution with n–1 degrees of freedom. The total error of individual sample measurements is slightly higher because of the addition of the nonlinearity correction error of δ ^34^S and the error from the calibration of both δ ^33^S and δ ^34^S, as shown below. The statistical analysis and all other analyses and creation of figures further described in this paper were performed using MATLAB R2020b.

***Nonlinearity***. Nonlinearity is the dependence of δ values on the integrated ion signal (peak area) of all isotope masses contributing to a certain peak in the IRMS chromatogram. Hattori *et al*. (2015) found a strong nonlinearity for their system between a COS total peak area of 0 and 8 Vs (mass 32, 33 and 34 combined), and they therefore decided to only measure total peak areas above 8 Vs. This meant, however, that a very large sample size of several hundreds of liters was required for a single measurement. For our measurements, in order to be able to measure smaller samples, the nonlinearity of the system was characterized and a correction factor to account for this nonlinearity was determined and applied to our data. This was done in two ways. For the first set of experiments, a 2 L glass flask (Norrmag, Ilmenau, Gerrmany with PCTFE sealing; Rothe *et al*., 2005) was filled with a gas mixture containing 2 ppb COS and zero air. The flask was connected on one side to the COS isotope measurement system and the other side of the flask was connected to a zero air cylinder. While keeping overpressure on the flask from the zero air cylinder, a series of 3 L measurements was conducted during which the gas mixture in the flask was more and more diluted with zero air with each subsequent measurement. The second method for characterizing the nonlinearity was done by injecting different volumes of the target gas, starting at 1 L and increasing in steps of 250 to 500 mL up to a volume of 4 L. In both methods, the dependence of the isotope values on the peak area was characterized by fitting a function through the data. From this trendline, a correction factor for the isotope values was calculated. The non-simultaneous 68% function bounds give the uncertainty of this function and thus the uncertainty added to the data when applying the nonlinearity correction.

The error from the nonlinearity correction and the reproducibility were combined following error propagation using Equation 3. This error represents the internal error, or how well individual measurements can be related to the internal lab scale.

$$\;\sigma_{total}=\sqrt{\sigma_{reprod}^{2}+\sigma_{nonlin}^{2}}\;(3)$$

***Calibration***. All measurements were first measured against the O _2_ working gas. Each sample was measured against our lab reference gas, which was a high-pressure cylinder, filled with ambient air at the surface of Greenland during the 2017 EastGRIP campaign ( Eastgrip, 2018). For the calibration against the international sulfur standard Vienna Canyon Diablo Troilite (VCDT), we used four COS calibration gases produced and calibrated at Tokyo Institute for Technology, which contained high mixing ratio COS (50 to 200 ppm) in nitrogen. The first calibration gas called “10.5 % COS” was a commercially obtained COS gas in high-purity He (99.99995 % purity; Japan Fine Products Co. Ltd., Kawasaki, Japan). The other three calibration gases were synthesized as described in Hattori *et al*. (2015) from three kinds of sulfur powders: Wako, Sigma-Aldrich and a mixture of the two. The synthesized COS gases were prepared at Tokyo Institute of Technology through a reaction of the sulfur powders and CO gas (99.99 % purity; Japan Fine Products Co. Lt., Kawasaki, Japan) in glass tubes at 573 K for 24 hours. The gases were purified using a GC equipped with a thermal conductivity detector (GC-14B; Shimadzu, Kyoto, Japan) and a packed column (Porapak Q, 2 mm i.d. 2.4 m; GL Science, Tokyo, Japan) maintained at 333 K.

The above-mentioned calibration gases were assigned δ ^34^S values on the VCDT scale by both an off-line and on-line method performed in the Geo Science Laboratory in Nagoya, Japan. For the off-line method, 20 μmol of each COS gas was reacted with an alkaline zinc solution (zinc acetate NaOH), and the sulfur was precipitated as ZnS overnight. The ZnS was washed by adding 18 MΩcm water (ZRXQ005T0, MerckMillipore, Corp., Burlington, MA, USA) and centrifuging, three times. The ZnS was combusted to SO _2_, of which the δ ^34^S values were measured on the VCDT scale by elemental analyzer (EA)-IRMS. The total error (1 σ) derived from repeatability and accuracy was 0.4 ‰ for the off-line method. The remaining COS gases were pressurized with high-purity nitrogen (99.99995 % purity; Japan Fine Products Co. Ltd., Kawasaki, Japan) to 1067 mbar and were stored in 3 L SilcoCan canisters (27303, Restek Corp., Pennsylvania, USA) for the on-line measurement. The on-line measurements were performed by Tokyo Institute of Technology according to the method developed previously ( Hattori *et al*., 2015; Kamezaki *et al*., 2019). The total error (1 σ) derived from repeatability, size dependence and accuracy was 0.3 ‰ for the on-line method. The δ ^34^S _VCDT_ values of the on-line and off-line measurements were very similar. The off-line values were used for fitting the calibration line, which yielded a calibration range for δ ^34^S of –8.9 ‰ to 13.3 ‰.

The calibration of δ ^33^S of these same calibration gases on the VCDT scale was done by calculating their values relatively to the δ ^33^S of a calibration gas (11 ppm COS) that has been used for δ ^33^S calibration in the past by Kamezaki *et al*. (2019). This method assumes that the δ ^33^S of this calibration gas has not changed since it was last measured in 2017. This assumption is supported by the fact that the δ ^34^S value of this same gas has been confirmed several times between 2017 and the present and no drift has been found. A mass-dependent relationship between δ ^33^S and δ ^34^S was found for all other calibration gases in the past, thus we assume that no mass-independent drift has occurred in the “11 ppm OCS” calibration gas. The calibration range for δ ^33^S is –7.8 ‰ to 6.6 ‰.

During our calibration procedure, the high COS mixing ratio calibration gases were diluted into 6 L canisters using zero air to a mixing ratio of 30 to 50 ppb. Two of the gases were chosen for further dilutions, which were the ones that had isotopic compositions that were most different from ambient air. These gases were diluted further in 5 L cylinders to a pressure of 100 bar, and a mixing ratio of approximately 700 ppt. During the calibration procedure we performed measurements of different volumes of all the dilutions made from the initial gases. We measured at different total peak areas between 1 and 6 Vs, to check for any nonlinearity effects.

All measurements were combined to derive a calibration curve, where the assigned δ ^33^S _VCDT_ and δ ^34^S _VCDT_ values from Japan are plotted against the δ ^33^S and δ ^34^S values measured in Utrecht, relatively to our reference gas. Trendlines were fitted using a linear regression method ( York *et al*., 2004), which considers the errors in both the X and Y direction. A slope of the calibration line >1 was considered as an evidence of scale contraction and, if needed, a correction method was developed. The results of the calibration procedure are presented below. The 68% uncertainty bounds were calculated using a bootstrapping resampling procedure to calculate the calibration error, which indicates how well the measurements in Utrecht can be linked to measurements in other labs on the VCDT scale.

***Data corrections, quality check and long-term stability***. At the beginning and end of each measurement sequence, blank measurements were performed, which were either 3 L injections of COS-free “zero air”, or “no-load” blanks in which no gas was injected into the pre-concentration system. The blank was found to be less than 5 % of the reference gas peak area and these blank measurement values were used to correct the peak area and isotope values of the measurements.

To monitor the long-term measurement stability, COS in air from a target cylinder (5 L, dried ambient air filled in Greenland in 2017 at 39m depth) was measured approximately weekly. A second target cylinder was introduced after the first gas cylinder was exhausted (ambient air high-pressure cylinder no. 1341). If an error occurred during the pre-concentration phase (e.g. incorrectly timed valve switching, sample not opened properly), or if a measurement looked clearly not good (e.g. bad GC separation or strange peak shape), those measurements were flagged and not included into the final dataset. In the end approximately 7 % of the measurements, including blank and reference gas measurements, had to be flagged due to the above-mentioned reasons.

***Additional tests***. This section describes some additional tests that were performed with the COS measurement system. These tests were performed to fully characterize the behavior of separate elements of the measurement set-up and to optimize the steps undertaken during the measurement procedure. The detailed results of these tests can be found in *Underlying data* ( Baartman *et al*., 2021).

Some materials are known or suspected to influence COS mixing ratios by either emitting COS or trapping it on their surfaces ( COSANOVA, n.d.). Therefore, several parts of the pre-concentration system were tested to make sure they did not affect our COS measurements. Firstly, the interference of the magnesium perchlorate dryer was tested, by comparing measurements of the same known gas with and without dryer. The influence of the vacuum pump at the end of the Tenax trap was also tested, by comparing measurements of the same gas with the pump turned on or off. No significant influences of the dryer or the vacuum pump were found.

Several tests were performed to optimize the release of COS from the Tenax trap. We investigated the effect of the heating temperature of the trap on the amount of COS that was released from the trap, in various ways. First, multiple COS isotope measurements were conducted using the same EastGrip 2017 gas cylinder but using different Tenax trap heating temperatures of 100 °C and 130 °C, where the measured COS peak areas were compared. Another test was to heat the Tenax in steps, increasing from room temperature to 200 °C with a 10 °C increase every 10 minutes, to see at which temperature the COS would be released. The effect of the duration of the Tenax trap heating was tested by comparing different heating durations to the COS peak area. The optimal heating procedure for COS release from the Tenax was 130 °C for 30 minutes.

The optimal time for flushing and heating the Tenax trap during the cleaning procedure in between measurements was also tested. This was done by performing 10 measurements with 15 minutes of heating and flushing and 10 measurements of the same EastGrip 2017 gas cylinder with 30 minutes of heating and flushing. The results were compared by checking the trend in COS peak areas within each measurement sequence of 10 measurements. An upward trend in peak areas of the measurements would point to an insufficient cleaning of the Tenax, leading to contamination of the next measurement. The optimal cleaning time for the trap was found to be 30 minutes.

The trapping efficiency of the Tenax trap was tested by placing a second Tenax trap after the first one and measuring a gas with ambient COS mixing ratio (target gas from EGRIP 2017). The COS that escaped the first trap would be trapped on this second trap. The traps were heated separately and the COS released from the trap could be measured independently. Tests were performed injecting volumes of air of 3 L, 4 L, 5 L and 6 L, to see if any breakthrough would occur at higher injected volumes. The trapping efficiency was found to be 100 % for all injected volumes that were tested.

Because of the presence of other compounds in the chromatogram ( Figure 2), we tested the possible interference of several available compounds on the trapping of COS, by measuring gas mixtures with known mixing ratios of 1.6 % CO _2_, 1 % CH _4_ and 4.5 % H _2_ and comparing these to measurements of gases without those gases present. CO _2_ interference was specifically tested since it was suspected to be one of the larger peaks on the chromatogram and since its possible interference was also mentioned by Angert *et al*. (2019). No interference of these compounds on COS trapping was found.

The memory effect and thereby also the blank peak area of the system was tested by first measuring a 3L injection of a gas with a COS mixing ratio of approximately 900 ppt, followed by a sequence of zero air measurements. By inspecting the peak area of the zero air measurements in the chromatogram, the memory effect was characterized. No significant memory was found with the current heating and flushing time of the Tenax, thus the influence of the previous sample measurement on the next is negligible.

### Ambient air measurements in Utrecht

Ambient air was drawn from outside the Buys Ballot building on the Utrecht University Campus (coordinates: 52.087471, 5.165394) with a sampling system that was directly connected to the pre-concentration system, as indicated in Figure 1 in red. The sampling system consisted of a ¼” Dekabon tube, which ran through a small hole in the wall of the lab to the outside. The opening of the tube was about one meter from the building wall, and at an elevation of approximately 15 m from the ground (on the 6 ^th^ floor). A magnesium perchlorate dryer was installed at the end of the sampling tube and was replaced regularly while sampling. The air was drawn in using a small membrane pump (type PM22874-86, KNF, France), which created a continuous flow rate of around 2 L min ^-1^. When sampling air, 80 mL min ^-1 ^was split into the pre-concentration system, while the rest of the air was vented. The pressure of ambient air going into the pre-concentration system was regulated by a needle valve and a flow controller at the outlet of the sampling system. Setting up the system in this way allowed for a continuous high flow through the Dekabon tube, so that there would be no stagnant air in the tube, and therefore less chance of contamination. One measurement with this set-up, including flushing and cleaning time of the Tenax trap, takes three hours.

Because the ambient air was running through the KNF pump before entering the pre-concentration system, we tested the possible interference of this pump on COS mixing ratios and isotopic composition. This was done by connecting one of the target gas cylinders to a 2 L glass flask, and connecting the sampling pump to the other end of the flask. The glass flask was added to create a volume of air between the pressurized cylinder and the pump, to prevent harm to the cylinder regulator. The outlet of the pump was then connected to the multi-position valve of the pre-concentration system. Using this set-up, we measured the same injected volume of the same gas 10 times. The results of this test were then compared to 10 measurements of the same gas with the same set-up, but with the pump removed. No significant effect of the pump on the measurement results was found.

A series of 15 sequences of COS isotope measurements from ambient air were performed between mid-October 2020 and January 2021, which yielded a total of 80 individual outside air measurement points. Each sequence consisted of four up to 12 ambient air measurements, interspersed with reference gas measurements.

Backward trajectory analysis using the Hybrid Single-Particle Lagrangian Integrated Trajectory model ( HYSPLIT) was performed in order to determine the prevailing wind directions during sampling and the main air origins ( Stein *et al*., 2015). The backward trajectories were calculated going back 96 hours from the time of the last sampling, with a new trajectory being calculated every 2 hours during the measurement period for an elevation of 20 m.

### Highway tunnel measurements

The Utrecht University campus is situated close to a busy highway and some highway junctions. As it is reported that cars do emit small amounts of COS both by combustion and tire wear ( Lee & Brimblecombe, 2016; Zumkehr *et al*., 2018), this highway could be a potential local source influencing our ambient air measurements. In order to assess the possible influence of traffic emissions, we collected some samples from a highway tunnel in the Utrecht region. Samples were taken in the Leidsche Rijntunnel (52°05’09.6” N 5°04’32.2” E), which has a length of 1650 m, a speed limit of 100 km/h and consists of four separated unidirectional tubes. The inner tubes have three driving lanes, while the outer have two lanes. The average traffic intensity for this tunnel was 200.000 vehicles per 24 hours ( Rijkswaterstaat, 2020). We drove with a 2012 Volkswagen transporter, equipped with a cavity ring-down analyzer, Picarro Inc. G2301. The G2301 was able to measure mixing ratios of CO _2_, CH _4_ and H _2_O. For a full description of the van and the analyzers, see Maazallahi *et al*. (2020), who used the same set-up for mobile CH _4_ measurements.

The samples for COS analysis were collected in pre-evacuated 6 L ENTECH Silonite canisters as follows: a 1.5 m Dekabon tube was connected to the outside of the van and sticking 1 m upwards from the top of the van door. A magnesium perchlorate dryer was installed between the tubing and the canister, which was replaced every two canisters. When sampling, the canister was opened a couple of seconds after entering the tunnel and closed again some seconds after exiting. An air inflow was maintained all throughout the sampling in the tunnel, however, with the canisters being evacuated at the start, the samples could have been slightly biased to the air at the entrance of the tunnel. The resulting pressure in the canisters was slightly below atmospheric as no pump was used during the sampling procedure. A total of six canisters was collected during the six tunnel drive throughs, of which one in the inner tube of the tunnel and the other 5 in the outer tubes. One day after sampling, the samples were diluted with COS-free synthetic air to increase the pressure of about 1.7 bar and measured with the COS isotope measurement system. Besides the online measurements, the samples were also measured for CO _2_ and CH _4_ mixing ratios, using the above mentioned Picarro G2301 instrument.

For interpretation of the COS isotope and concentration data, Keeling plots ( Keeling, 1961; Pataki *et al*., 2003) were created using MATLAB R2020b.

## Results and discussion

### Reproducibility

**Figure 3.**
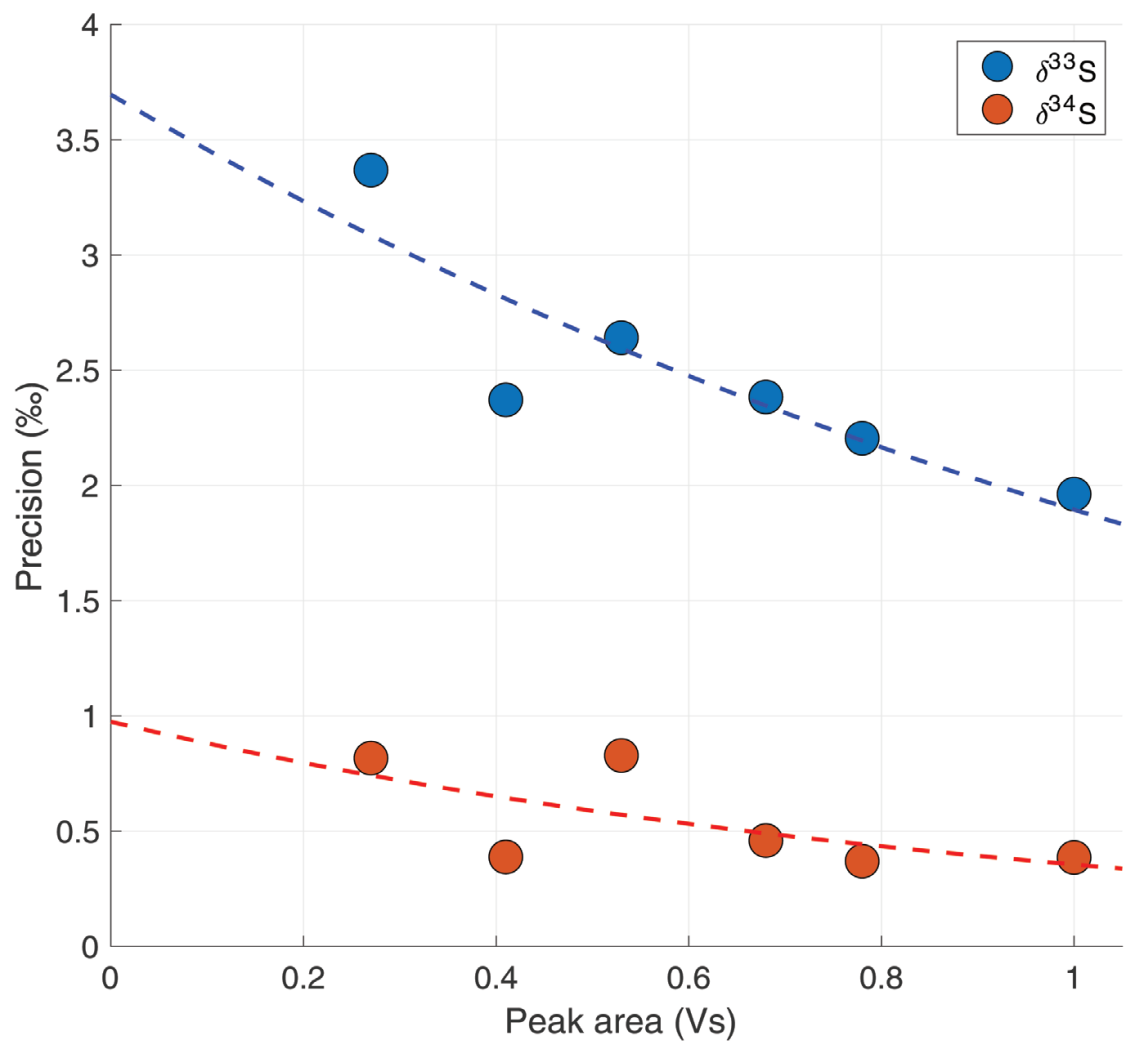
The precision in ‰ as a function of total peak area in Volt seconds (Vs). Every data point represents the 70% confidence interval from a student t-distribution of 6 – 8 measurements combined.

Figure 3 shows the measurement reproducibility as a function of the measured COS peak area (n = 6 to 8), where each dot in the scatter plot represents the precision of a set of measurements. A trendline was fitted through the data-points to use as a continuous function for the precision when calculating the error for individual samples. The dependence of the precision on peak area, within the range of our measurements (peak area between 1 and 0.2 Vs), can best be described by the exponential functions given in Equation 4 ( *R ^2^* = 0.76) and 5 ( *R ^2^* = 0.40).

$$\sigma_{\delta^{33}\text{S}}(A)=3.70e^{-0.67A}\;(4)$$

$$\sigma_{\delta^{34}\text{S}}(A)=0.97e^{-1.01A}\;(5)$$

Where σ is the reproducibility error in ‰, and A is the peak area of a measurement. For reference, a measurement of 4L of ambient air with an ambient mixing ratio of 550 ppt will give a peak area of approximately 0.68 Vs, which would correspond to a system error of 2.1 ‰ for δ ^33^S and 0.4 ‰ for δ ^34^S.

### Nonlinearity

**Figure 4.**
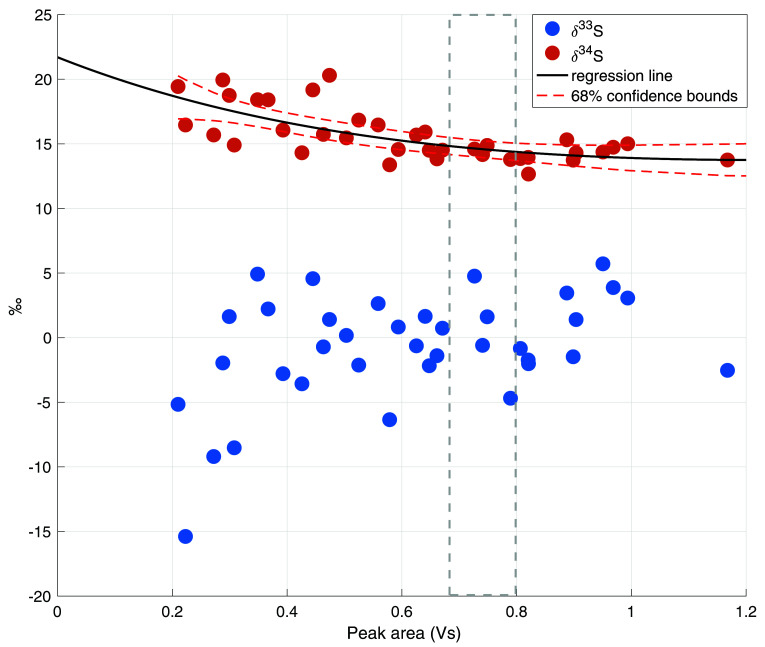
Nonlinearity plot for both δ ^33^S and δ ^34^S, with the total peak area in Volt seconds (Vs) on the x axis and the isotope values in ‰ on the y axis. The black line is the linear regression line for δ ^34^S, including the 68% confidence bounds in red dashed lines. The grey dashed lines indicate the usual peak area for a 4 L ambient air measurement.

Figure 4 shows the nonlinearity data including a regression line and the 68% functional bounds for the δ ^34^S data. It can be observed that the nonlinearity effect for δ ^34^S is minor and only a small correction was needed for the ambient air measurements, which had peak areas between 0.65 and 0.8 Vs (indicated with the shaded area in Figure 4). Equation 6 and 7 describe the correction made for δ ^34^S with a peak area smaller than 1 Vs, where A _ref_ is the peak area of the reference measurements and A _sample_ is the sample peak area. For δ ^33^S, the nonlinearity effect only starts to be evident from peak areas smaller than 0.4 Vs. Since our dataset does not include any measurements with such low peak areas, no correction for δ ^33^S was applied.

$$\delta^{34}S_{correction}=[5.17e^{-0.49A_{ref}}+9.66e^{0.1A_{ref}}]-[5.17e^{-0.49A_{sample}}+9.66e^{0.1A_{sample}}]\;(6)$$

$$\;\delta^{34}S_{corrected}\left(A_{sample}\geq 1\right)=\delta^{34S}\left(A_{sample}\geq 1\right)+\delta_{correction}^{34S}\left(A_{sample}\geq 1\right)\;(7)$$

### Calibration

**Figure 5.**
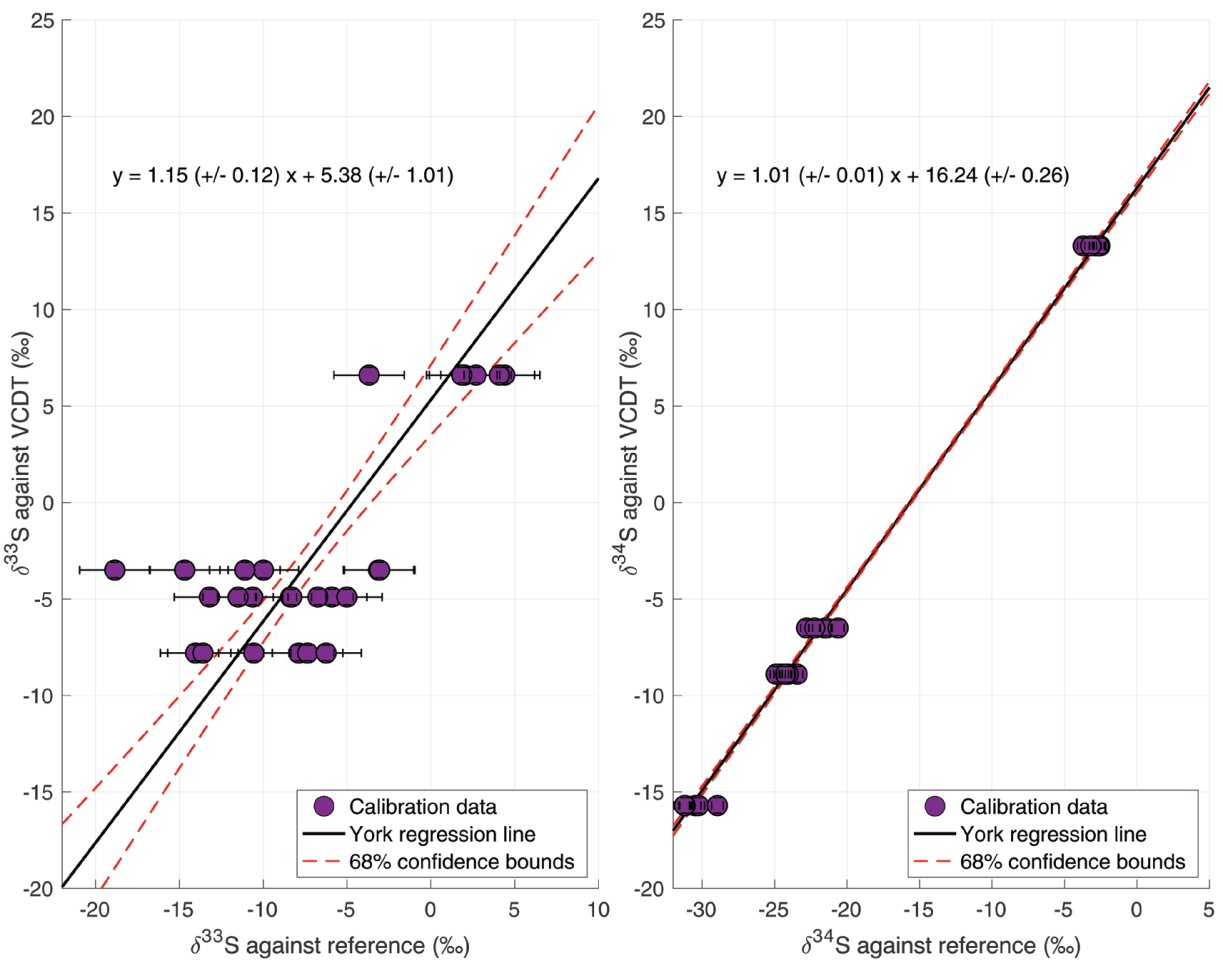
Results of the isotope calibration of δ ^33^S and δ ^34^S based on calibration gases provided by Tokyo Institute of Technology. The data points represent the individual calibration measurements. The black line is the York regression line ( York *et al*., 2004), and the red dashed lines represent the 68% confidence bounds of the regression line.

The results of the calibration are available in *Underlying data* ( Baartman *et al*., 2021). Table 1 shows the results of the calibration with the four calibration gases: Wako, Sigma-Aldrich, Mix (a mixture of Wako and Sigma-Aldrich) and 10.5 % COS. Figure 5 shows the assigned δ ^33^S _VCDT_ and δ ^34^S _VCDT_ values of the calibration gases against the results of our measurements relative to the reference gas. These were used to calculate the calibration functions for our measurements, also shown in the figure.

**Table 1.** Results of the COS calibration with four calibration gases “Wako”, “Sigma-Aldrich”, “Mix” and “10.5 % OCS”, where δ ^34^S Utrecht and δ ^33^S Utrecht are the isotope values measured by our measurement system in Utrecht, δ ^34^S _vcdt_ off-line are the δ ^34^S values measured by the off-line method in Japan, δ ^34^S _vcdt_ on-line are the δ ^34^S values measured by the on-line method in Japan and δ ^33^S vs “11 ppm OCS” gas are the δ ^33^S values on the VCDT scale calculated relatively to the δ ^33^S of a calibration gas called “11 ppm OCS” that was used previously for the δ ^33^S calibration by Kamezaki *et al*. (2019).

Gas	δ ^34^S _ref-Utrecht_	δ ^34^S _vcdt_ off-line	δ ^34^S _VCDT_ on-line	δ ^33^S _ref-Utrecht_	δ ^33^S vs “11 ppm OCS” gas
Wako	-24.3 ± 0.4	-8.9 ± 0.4	-9.3 ± 0.3	-8.7 ± 2.9	-4.9 ± 0.4
Sigma-Aldrich	-21.8 ± 0.8	-6.5 ± 0.4	-6.1 ± 0.3	-10.1 ± 6.3	-3.5 ± 0.4
Mix	-30.7 ± 1.2	-15.7 ± 0.4	-15.0 ± 0.3	-10 ± 3.3	-7.8 ± 0.4
10.5 % COS	-2.9 ± 0.4	13.3 ± 0.4	13.5 ± 0.3	1.9 ± 2.9	6.6 ± 0.4

For δ ^34^S, the slope of the calibration line is 1.01 ± 0.01, which means there was no significant scale contraction and no correction was necessary. The calibration gases gave us a broad calibration range, from δ ^34^S = –15.7 ‰ to +13.3 ‰ VCDT, bracketing the atmospheric δ ^34^S values. For δ ^33^S, a slope of 1.15 ± 0.12 was found, which means that there may be some small-scale contraction effect for δ ^33^S and a correction was applied. Furthermore, a larger spread in the measurement data for δ ^33^S lead to a larger error of the calibration line (red dotted lines in Figure 5), and to a substantially larger total error. One of the calibration gases (gas 2, Sigma-Aldrich) showed a larger spread in the results for both isotopes, which increased the uncertainty in both calibration lines, but especially the calibration line of δ ^33^S. When combining the errors from the reproducibility, the nonlinearity correction and the calibration, the total error for a 4 L ambient air sample is 3.3 ‰ for δ ^33^S and 0.9 ‰ for δ ^34^S.

***Additional tests***. The magnesium perchlorate dryer did not have a significant effect on the amount of COS trapped in the Tenax trap nor on the isotopic composition of the measurements. There was no significant difference in mean peak area or δ ^33^S and δ ^34^S with and without the dryer. Because the presence of water in the system could be detrimental to the pre-concentration system, we decided to keep a dryer the line before the Tenax trap for all measurements.

 The membrane pump at the end of the system also did not significantly change the peak area nor the isotopic composition of the COS measurements. This pump allowed us to measure at higher flows and measure from samples until lower pressures, thus the pump was included for all measurements.

 The optimal heating temperature of the Tenax for COS release to the focus trap is 130 °C. This temperature yielded the highest peak area for the same amount of injected gas, and when heating to higher temperatures, no more COS was released from the trap. It was found that most of the COS was already released at room temperature, which was also noticed by Tangerman (1986). The optimal cleaning time of the trap in between measurements was found to be 30 minutes. During the Tenax trapping efficiency test with the second Tenax trap we found that the peak area of COS released from the second trap was equal to blank measurements. This test was performed up to volumes of 6 L of injected air. In all tests, the Tenax trap had a >99 % trapping efficiency.

The presence of CO _2_, CH _4_ or nitrogen did not have an influence on the trapping efficiency or isotopic composition of the measured COS, which is in agreement with similar tests performed by Angert *et al*. (2019).

When testing the memory effect in the system, we found that the peak area of the first zero air measurement after the sample measurement was 5.1 % of the sample measurement peak size. The peak area did not decrease much in the subsequent zero air measurements. By the 9th measurement, the peak area had decreased only to 4.9 % of the sample peak size. The other type of blank measurements, where no sample was loaded at all, also yielded a peak area between 2 and 5 % of the sample measurement peak area. We therefore assumed that the interference of the previous gas on the next measurement was minimal, and that our system has a constant 5 % blank level. However, we made sure to always measure a 3 L zero air injection after a measurement of a gas with higher COS mixing ratio, in order to minimize a potential memory effect on the subsequent measurement.

### Long term stability of the measurement system

**Figure 6.**
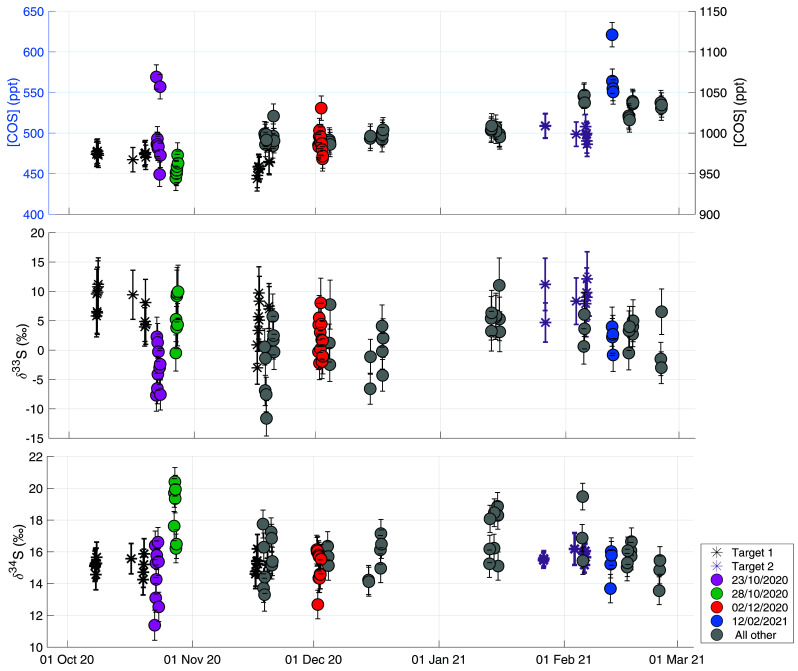
Ambient air time-series plotted together with measurements of two target gases in black and purple asterisks, to show the long-term variability of the measurement system. The second target gas (purple asterisks) was introduced after the first one was exhausted, and has slightly higher mixing ratio than the first target gas (black asterisks). Upper figure shows COS mixing ratio with the ambient air measurements plotted on the left y-axis and the target measurements on the right y-axis. Middle figure shows δ ^33^S, and the lower panel shows δ ^34^S. All data are plotted against date and time on the x-axis. some sequences of special interest are highlighted in the colors pink (Oct 22 – Oct 23), green (Oct 27 – Oct 28), red (Dec 1 – Dec 2) and blue (Feb 12). The grey colors represent all other measurements.

Figure 6 shows the time series of online ambient air measurements with the target gas measurements plotted as grey and purple asterisks in the background. Target measurements were performed throughout the measurement period in order to monitor long-term variability and characterize potential drift in the system. The two different target gases are indicated as different color asterisks in the figure. It can be observed that for the COS mixing ratio there is little variability in these target measurements on the daily scale and also no evidence of drift on the longer time-scale. The variations in COS mixing ratios of the ambient air measurements are clearly larger than in the target measurements. For δ ^34^S, the day-to-day variability of the target is up to a maximum of 2 ‰ and there is no evidence for drift visible on longer time-scales. The error of the target measurements over the course of the measurement period was 0.5 ‰ (1 σ). The day-to-day variability of the target measurements is larger for δ ^33^S; from 5 ‰ up to around 12 ‰, indicating that measurements of δ ^33^S with our system are less stable and we should be careful with drawing conclusions on small variations of δ ^33^S in sample measurements. However, also for δ ^33^S there is no apparent long-term drift in the target measurements, and the error of the target over the measurement period was 3.4 ‰ (1 σ), which is similar to the total error for an ambient concentration 4 L air sample.

### Ambient air measurements Utrecht

**Figure 7.**
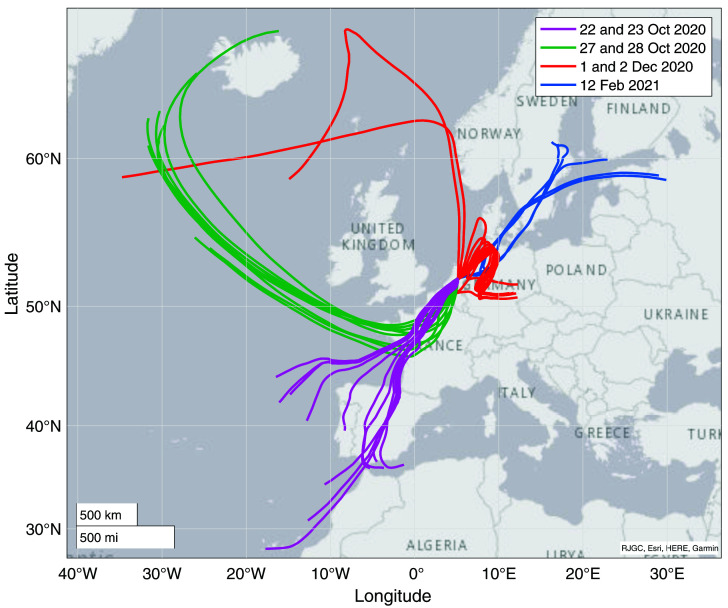
Results from backward trajectory modelling using HYSPLIT ( Stein *et al*., 2015). The colors correspond to the measurement days, highlighted with the same colors as the data points in Figure 7.

The Utrecht ambient air dataset is available in *Underlying data* ( Baartman *et al*., 2021). Figure 6 shows all air measurements, and, indicated with different colors, measurement sequences of special interest that correspond to the HYSPLIT backward trajectory results shown in Figure 7. The trajectories for all the measurement days in the time-series can be found in *Underlying data*. The mean COS mixing ratio during the measurement period was 503 ± 15 ppt, with a variation between 450 and 650 ppt. An increasing trend was observed in the transition from the fall to the winter season, from a mean of 488 ± 15 ppt in fall to 530 ±15 ppt in winter. This trend is to be expected as the biosphere is the largest sink of COS and becomes mostly inactive during winter in the Northern Hemisphere ( Montzka *et al*., 2007). The maximum observed mixing ratio observed was 621 ppt on Feb 12 ^th^ 2021. The mean δ ^33^S and δ ^34^S over the measurement period were 1.1 ± 3.3 ‰ VCDT and 15.7 ± 0.9 ‰ VCDT respectively. We estimate the background δ ^33^S and δ ^34^S values in Utrecht by assuming a background COS mixing ratio between 480 and 510 ppt and selecting the mean δ values ± 1 σ from this background mixing ratio selection. Using this method, we obtain background values of 1.0 ± 3.4 ‰ (μ ± SE) and 15.5 ± 0.8 ‰ (μ ± SE) for δ ^33^S and δ ^34^S respectively. Our background value for δ ^34^S value is slightly higher than the numbers that have been reported in previous studies. Davidson *et al*. (2021) found a mean tropospheric δ ^34^S of 13.9 ± 0.1 ‰ and Hattori *et al*. (2020) estimated a background δ ^34^S of 12 to 13.5 ‰ for Japan. This higher background δ ^34^S and also the lack of very high COS mixing ratios in our measurements from Utrecht could possibly be explained by a lower amount of anthropogenic emissions in this region, but could also be due to a larger influence of the biosphere sink that enriched northern hemisphere air masses during summer and fall ( Davidson *et al*., 2021), or a larger contribution of the ocean source. A cross-calibration of measurements between different measurement laboratories would be useful to investigate whether differences in measured background δ ^34^S are significant atmospheric signals or are still due to measurement uncertainty. The value for δ ^33^S of 1.0 ± 3.4 ‰ is lower than expected from mass-dependent processes. Equation 8 presents the calculation of Δ ^33^S, which describes the deviation of δ ^33^S from the mass-dependent fractionation line ( Farquhar & Wing, 2003; Ono *et al*., 2006). Δ ^33^S would in this case be slightly negative, however, as there is still a substantial uncertainty in our calibration of the δ ^33^S measurements, we should be careful with the interpretation of these values.

$$\Delta {\;}^{33}S=\delta {\;}^{33}S-[(\delta {\;}^{34}S+1){\;}^{0.515}-1]\;(8)$$

While there are some short-time variations in δ values, there is no significant seasonal trend visible in δ ^34^S, with a mean δ ^34^S value in the fall (22 October – 22 December) of 15.8 ± 0.9 ‰ and 16.2 ± 0.9 ‰ in winter (22 December onwards). Davidson *et al*. (2021) found a seasonal variation in δ ^34^S between spring and fall of 1.2 ‰, which was ascribed to the effect of a small fractionation during plant uptake of –1.9 ‰. As our time-series does not include the spring and summer seasons yet, and as the seasonal trend is expected to be small, it is not surprising that we do not observe such a seasonal trend in our dataset.

**Figure 8.**
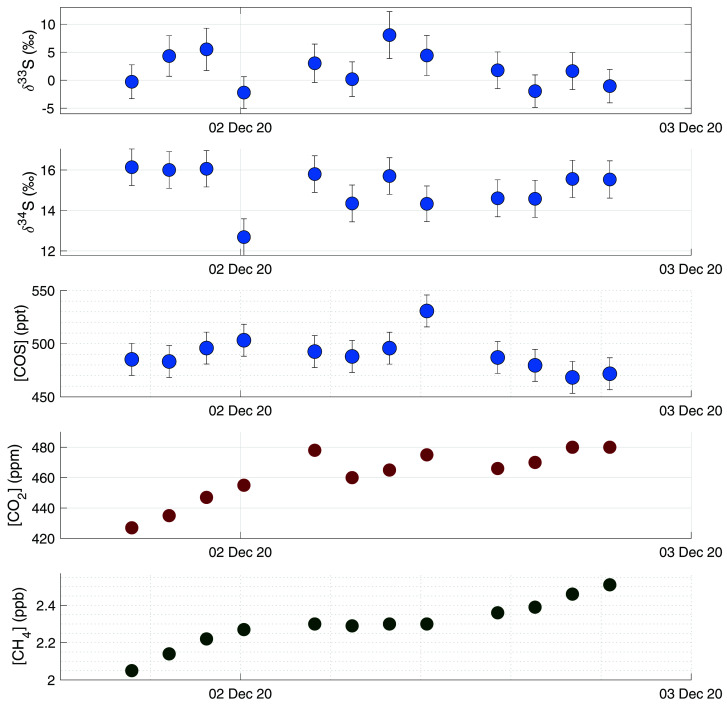
Results from a measurement sequence on the 1 ^st^ and 2 ^nd^ of December, during which a pollution plume advected from Germany was observed. Figures from high to low: δ ^33^S, δ ^34^S, COS mixing ratio, CO _2_ mixing ratio and CH _4_ mixing ratio.

 More information can be gained from this dataset when looking more closely at the variability on the day-to-day to weekly scale, and a comparison to the backward trajectory analyses shown in Figure 7. Potentially interesting are the days that were influenced by anthropogenic industrial emissions. Hattori *et al*. (2020) reported large enhancements in COS mixing ratios, accompanied by depleted δ ^34^S values, when measuring air from the Chinese mainland. It is likely that in Europe there is less anthropogenic COS emission, but no measurements have been presented so far on these emissions nor has their sulfur isotopic composition been reported. On the 1 ^st^ and 2 ^nd^ of December, the Netherlands experienced mostly Easterly winds, bringing in polluted air from the Ruhr area, a large industrial region in Western Germany. COS data from these days are shown separately in Figure 8, together with CH _4_ and CO _2_ mixing ratios. During these days, CO _2_ and CH _4_ mixing ratios increased, with a maximum CO _2_ mixing ratio of 480 ppm and CH _4_ mixing ratio peaking at 2.5 ppb. During the first hours of this pollution accumulation, COS mixing ratios also increased from around 480 to 520 ppt at the maximum. The ratio COS/CO _2_ for these first four measurements was approximately 0.7 ppt/ppm. However, while during the evening of the 2 ^nd^ of December both CO _2_ and CH _4_ mixing ratios continued to increase, COS mixing ratios decreased again to below 480 ppt. During this period, we see small variations in both δ ^33^S and δ ^34^S, with some depleted values for the highest mixing ratios, but the differences are generally small. Thus, while there was a large increase in both CO _2_ and CH _4_ mixing ratios, we did not observe a substantial increase in COS mixing ratio, nor was there a large trend in δ ^33^S or δ ^34^S values. We can therefore conclude that this pollution plume did not contain large amounts of COS, and this major industrial area in Germany may not contribute much to the global anthropogenic COS emissions. More generally, the impact of European anthropogenic emissions on the COS budget in the Netherlands is likely small, because we generally do not see events with high COS mixing ratios and/or very depleted values of δ ^34^S during our measurement period. Looking at the gridded global anthropogenic inventory data of Zumkehr *et al*. (2018), there is some emission visible in western Europe, and indeed also in the Ruhr area. However, when comparing the European anthropogenic COS emission to that of East Asia, there are fewer sources, and also lower COS emissions from these source locations. Zumkehr *et al*. (2018) also state in their paper that 45 % of the global anthropogenic COS emissions come from China, and the rest of the emissions are relatively evenly spread over India, North America and Europe. This would explain the absence of large COS enhancements during our measurement period compared to previous studies by Hattori *et al*. (2020), who measured enhancements in air originating from China.

 Analysis of air origins through backward trajectory modelling provides insight into the regional sources and sinks of COS. The Netherlands is situated in the mid-latitudes and receives air from very different origins, which can change the influence of different sources and sinks on the COS that we measure in Utrecht. When we for instance look at the data subset of the 23 ^rd^ of October and compare it to the 28 ^th^ of October, we see large differences in both COS mixing ratio and δ values. The backward trajectories of these two days, indicated with pink (22 and 23 Oct) and green (28 Oct) in Figure 7, show that on Oct 23 ^rd^, the air came mostly from the South and travelled far over the continent across Spain and France at an altitude of just below 500 m, before reaching Utrecht. On Oct 28 ^th^, the air originated from the North and travelled mostly over the ocean at low altitude of mostly below 200 m, according to the HYSPLIT trajectories. On Oct 28th we see a lower and more stable COS mixing ratio of 455 ± 9.5 ppt than on the 23 ^rd^, which had a mean mixing ratio of 509 ± 39 ppt. On the 28 ^th^, we also observe high mean δ values of 5.3 ± 3.3 ‰ and 18.9 ± 1.2 ‰ for δ ^33^S and δ ^34^S respectively. In contrast, on the 23 ^rd^ we find mean δ values of –2.7 ± 3.1 ‰ and 14.0 ± 1.2 ‰ for δ ^33^S and δ ^34^S respectively. One explanation for these differences could be that on land there are more sources and sinks influencing COS (being biosphere uptake and anthropogenic emissions), which would create more variability in the mixing ratio; a phenomenon we see on the 23 ^rd^. On the 28 ^th^ we probably see a representation of rather clean background air, probably affected by ocean emissions, which is proposed to have a value of 19 ‰ ( Angert *et al*., 2019; Davidson *et al*., 2021; Hattori *et al*., 2020), which is higher than the background. Biosphere uptake in the higher latitudes could be a stable sink decreasing the mixing ratio and due to fractionation effects also making the COS more enriched in ^34^S ( Davidson *et al*., 2021). However, quantification of these sources and sinks requires more observations and the use of models.

**Figure 9.**
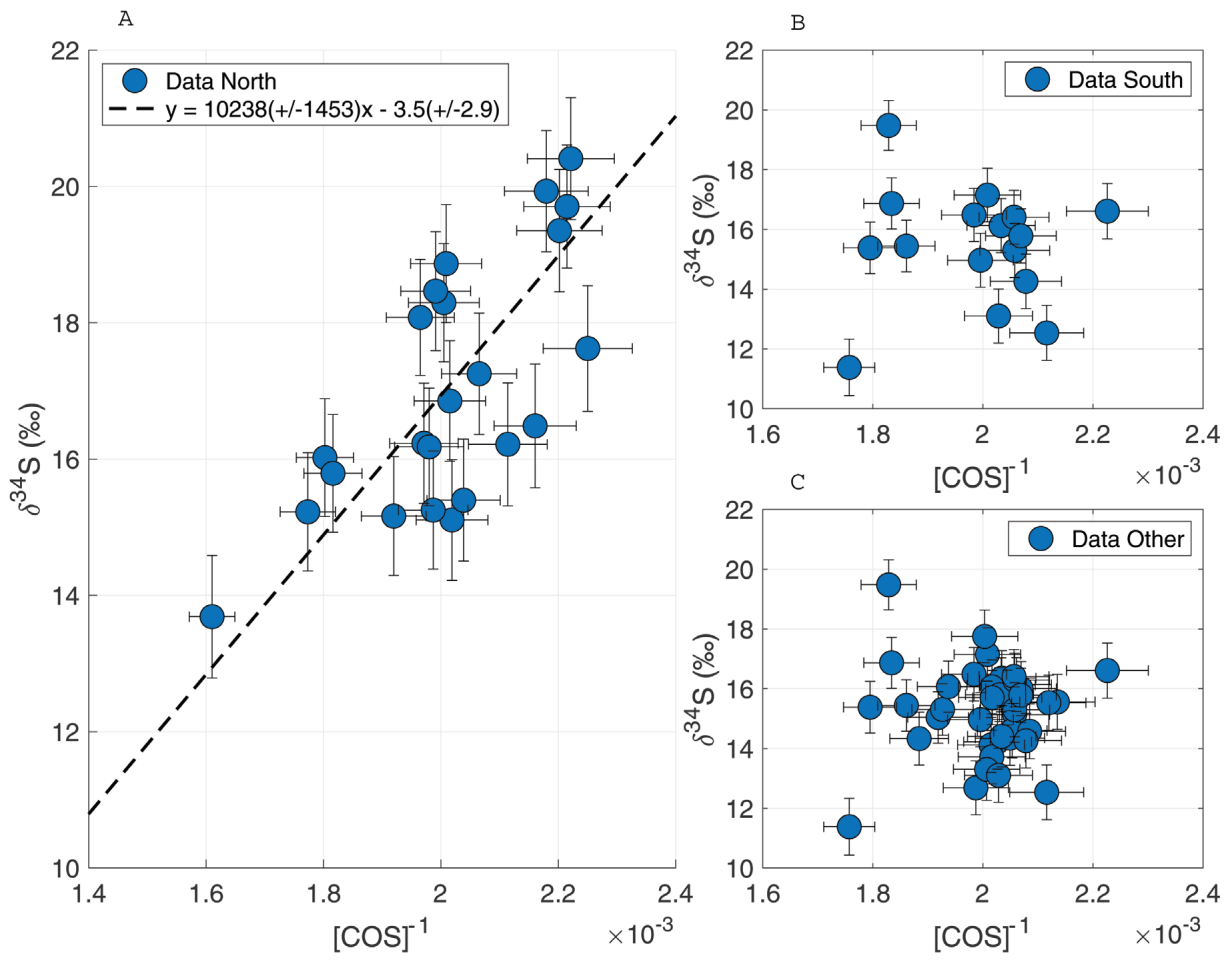
Keeling plots for δ ^34^S for data subsets from different wind directions. **A**: wind direction from the North, **B**: wind direction from the South, **C**: all data excluding wind direction from the North.

 To gain more insight into the processes influencing the COS from different air origins, the following data subsets were created: only northern air, only southern air and all “other directions” air. Relatively high mixing ratios were found in the northern air data subset, thus Keeling plots were created of the data subsets to assess whether there were any trends present and whether source signatures could be identified. As can be seen in Figure 9, a dependence of the δ values on the mixing ratio can only be found for the northern air data. This Keeling plot shows a picture that is consistent with an isotopically depleted COS source, with a Keeling plot intercept of –3.5 ± 2.9 ‰. A potential ^34^S depleted source could be anthropogenic COS, however, previous estimates of δ ^34^S from anthropogenic COS were slightly higher than this Keeling intercept ( Davidson *et al*., 2021; Hattori *et al*., 2020). While there are some large industrial areas in in the North of the Netherlands, more local measurements are needed to confirm this rather depleted anthropogenic source. On the low concentration end of the Keeling plot, COS is enriched in ^34^S, which could be due to fractionation taking place during biosphere uptake in higher latitudes.

### Highway tunnel measurements

The highway tunnel dataset is available in *Underlying data* ( Baartman *et al*., 2021). While driving through the several tunnel tubes, the Picarro analyzers clearly measured an increase of CO _2_ from the entrance to the exit of the tunnel, indicating a build-up of exhaust gases inside all the tunnel tubes. The on-line CO _2_ mixing ratio data can be found in *Extended data* ( Baartman *et al*., 2021). Besides CO _2_, no clear elevation of the other compounds (CH _4_ and H _2_O) was measured. From the total of six samples taken in the tunnel for COS measurements, two samples were not suitable for isotope measurements as they had too low pressure and therefore, we only report the results of four tunnel samples.

The results of our sample measurements show elevated COS mixing ratios in the tunnel, with a maximum increase of around 50 to 160 ppt compared to the background mixing ratio of 490 ppt. The maximum mixing ratio in a sample taken inside the tunnel was around 650 ± 15 ppt. The CO _2_ mixing ratio in the samples was between 541 and 606 ppm. From these values we calculated the COS/CO _2_ enhancement ratio, which was 0.4 ± 0.08 ppt/ppm for the first three samples and 1.15 ± 0.1 ppt/ppm for the last sample. As the first three samples had such a consistent COS/CO _2_ ratio, we assumed that the fourth sample was possibly contaminated by a single high COS-emitting vehicle, and we excluded it from the ratio estimation. If we assume that this ratio of 0.4 ppt/ppm is typical for a European fleet, we can make a very rough estimate of the annual European COS emissions from road traffic. Using the annual CO _2_ emission from road transport (sector name 1.A.3b) for the year 2018 ( European Environment Agency, 2020) of around 888 Tg CO _2_, we find a COS emission of 0.19 Gg S a ^-1^. Earlier estimates of global COS emissions from road traffic are in the range of 0.8 – 8 Gg S a ^-1^ ( Chin & Davis, 1995; Fried *et al.*, 1992; Watts, 2000; Lee & Brimblecombe, 2016) with the most recent estimate being on the higher side of this range with 6 ± 4 Gg S a ^-1^ ( Watts, 2000). Because the European Committee for Standardization (CEN) fuel content standard states a maximum sulfur content of only 10 ppm ( EEA, 2003) since the year 2009, we consider 0.19 Gg S a ^-1 ^for Europe a reasonable estimate. However, as our calculated COS/CO _2_ ratio is only based on a small dataset, this can only be seen as a rough first estimate of European COS traffic emissions.

**Figure 10.**
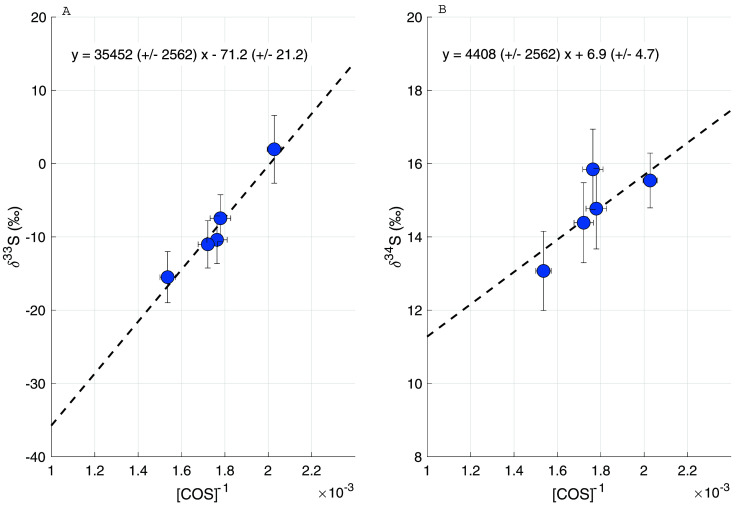
Keeling plot of the samples taken inside a highway tunnel, for δ ^33^S ( **A**) and δ ^34^S ( **B**).

Figure 10 shows the Keeling plots for δ ^33^S (left) and δ ^34^S (right), with the four tunnel samples and the average background value ± 1 σ uncertainty. A two-isotope plot of all the ambient air data and the tunnel sample data can be found in *Extended data* ( Baartman *et al*., 2021). The COS in the tunnel samples is depleted in both ^33^S and ^34^S with a Keeling intercept of –71.5 ± 21.2 ‰ for δ ^33^S and 6.9 ± 4.7 ‰ for δ ^34^S. In previous studies ( Angert *et al*., 2019; Davidson *et al*., 2021; Hattori *et al*., 2020), only the COS sulfur isotope ratios for total anthropogenic emissions have been reported, and a distinction between different anthropogenic sources has not been made thus far. The general trend of depleted values for δ ^34^S for traffic emissions roughly agrees with the current estimate for the anthropogenic emission signature of 3 to 8 ‰. The very low Keeling plot intercept for δ ^33^S of –71.5 ± 21.2 ‰ would indicate a strongly mass-independent process, which is unexpected, and it would be helpful to confirm this with additional studies. With the present evidence being based on only four samples, which were also processed slightly differently from ambient air (e.g. including a dilution step), we consider it premature to draw reliable conclusions. We note, however, that the sulfur in gasoline and diesel in Europe is highly modified by the complex sulfur removal process ( Srivastava, 2012) to reduce the sulfur content from % to ppm level ( EEA, 2003), which could in principle lead to anomalous δ ^33^S in fuel. If this low δ ^33^S is confirmed, strong processing might explain the unexpected sulfur isotopic composition of the COS emissions, although the mass-independent fractionation processes involved are still unexplained.

## Conclusion

In this paper, we described a new measurement system for δ ^33^S and δ ^34^S in COS, developed at Utrecht University, which enables measurements of small samples, with a relatively simple GC-IRMS system. A single measurement only takes 2 to 3 hours. We obtained a total precision for 4 L ambient air samples of 2.4 ‰ for δ ^33^S and 0.9 ‰ for δ ^34^S, when including the error in the nonlinearity correction and the calibration. The ability to measure small samples allows us to measure air from a wide variety of locations, which will allow us to characterize latitudinal and altitudinal variations in COS isotopologues.

The sampling system coupled to the pre-concentration system can measure ambient air in Utrecht with little maintenance effort. This will enable us to create a long-term record of COS mixing ratio and isotopic composition in the Netherlands, which will help to gain more insight in the seasonal and year to year variability of COS. The first results from ambient air measurement in Utrecht show a small increase in COS mixing ratio of 40 ppt from fall to winter. During the measurement period, no mixing ratios higher than 620 ppt were observed and the mean δ ^34^S of 15.9 ± 0.9 ‰ was relatively high compared to previously reported results by Angert *et al*. (2019), Kamezaki *et al*. (2019), Hattori *et al*. (2020) and Davidson *et al*. (2021). This leads us to conclude that the air in Utrecht likely receives relatively little COS from anthropogenic sources.

Three out of four measurements of samples taken inside a highway tunnel yielded a COS/CO _2_ ratio of 0.4 ppt/ppm, which can be extrapolated into a European estimate of COS from traffic emissions of 0.19 Gg S a ^-1^, which is in rough agreement with the current global estimates of COS emissions from traffic ( Chin & Davis, 1995; Fried *et al.*, 1992; Lee & Brimblecombe, 2016; Watts, 2000). The derived value of δ ^34^S = 6.9 ± 4.7 ‰ of traffic emissions is close to the reported values for anthropogenic emissions ( Angert *et al*., 2019; Davidson *et al*., 2021; Hattori *et al*., 2020). The very low value of –71.5 ± 21.2 ‰ for δ ^33^S in COS from traffic is unexpected and further measurements would be helpful to confirm this value. However, COS emissions from traffic make only a small contribution to the overall budget. Thus, more effort is needed to reduce uncertainties in the dominating sources and sinks in the COS budget using isotopic analysis.

## Data availability

### Underlying data

Zenodo: A GC-IRMS method for measuring sulfur isotope ratios of carbonyl sulfide from small air samples. https://doi.org/10.5281/zenodo.6319758 ( Baartman *et al*., 2021).

This project contains the following underlying data:

- COS_isotopes_ambient_air_data_Baartman_et_al.xlsx (dataset of ambient air measurements at Utrecht and highway tunnel).
- COS_isotopes_ambient_air_origin_subsets_data_Baartman_et_al.xlsx (dataset of the created subsets based on air origin from HYSPLIT model analyses).
- COS_isotopes_nonlinearity_test_Baartman_et_al.xlsx.
- COS_isotopes_reproducibility_Baartman_et_al.xlsx.
- COS_isotopes_target_data_Baartman_et_al.xlsx (target measurements dataset for the long-term stability).
- COS_isotopes_calibration_data_Baartman_et_al.xlsx (calibration data).
- HYSPLIT_backward_trajectories_Baartman_et_al.zip (folder with results from HYSPLIT backward trajectory analyses including .txt files and generated figures in .pdf).

### Extended data

Zenodo: A GC-IRMS method for measuring sulfur isotope ratios of carbonyl sulfide from small air samples. https://doi.org/10.5281/zenodo.6319758 ( Baartman *et al*., 2021).

This project contains the following extended data:

- Supplementary_figure1_Baartman_et_al.eps
- Supplementary_figure_2_Baartman_et_al.eps
- Supplementary_figure3_Baartman_et_al.eps

Data are available under the terms of the Creative Commons Attribution 4.0 International license (CC-BY 4.0).
