# Supplementary material for: A GC-IRMS method for measuring sulfur isotope ratios of carbonyl sulfide from small air samples
Source: Open Res Eur. 2022 Mar 8;1:105. Originally published 2021 Sep 13. [Version 2] doi: 10.12688/openreseurope.13875.2 (PMC10521041; doi:10.12688/openreseurope.13875.2)
Supplement: Peer review history

# Reviewer report: Reviewer response for version 1

**DOI:** 10.21956/openreseurope.14959.r27824

**Published:** 2021-10-27

**Authors:** Ralph Keeling

**Affiliations:** 1 Geosciences Research Division, Scripps Institution of Oceanography, University of California, San Diego, San Diego, CA, USA

This study describes a method for measuring changes in the isotopic composition of sulfur in atmospheric carbonyl sulfide (COS) and applies it to a few preliminary investigations. The method topic is skillfully introduced, the method mostly is described in admirable detail. I expect the method and preliminary results will be of general interest to the community working on COS and related measurements.

 I answered “partly” to the earlier question about data sources only because I did not try to fully assess the completeness of the supplemental data. For this study, readers will be most interested in reproducing the experimental method, not the calculations.

 I have only minor suggestions for improvement. As the submitted manuscript lacked line numbers, I’ve resorted to cutting a pasting from the text:

- “Direct measurements of GPP are difficult to obtain, because flux measurements only yield the sum the two almost cancel-ling fluxes: GPP and respiration.” Needs to mention CO _2_ fluxes.
- “The sulfur isotope ratios are reported as a δ values” Cut “a”.
- “Newman et al. (1991) presented the first global estimate of 34S in tropospheric COS of 11 ‰..” – The absolute value of 11 ‰ is ambiguous without a discussion of the standard used in Eq. (2). Could be fixed by mention the standard earlier when presenting Eq. (2).
- “The destruction reaction of COS with hydrogen oxide radicals (OH)” - Aren’t these more normally called ­hydroxyl radicals?
- “In short, the sample gas is first directed through a cooled Tenax trap..” I missed an explanation of why two cold traps at different temperature are needed. Why doesn’t the first trap achieve adequate focusing? Something to do with the materials in the trap and ability to warm quickly?
-  “After all the sample gas is injected” Context is not clear here. Injected where? What triggers stopping the sample flow?
- Figure 2: I see four peaks on the left, not three, so the caption is confusing. Are we to ignore the first peak? For clarity, I suggest labeling the peaks: O _2_, CO _2_?, COS, organics?
- “Calibration” Section starting page 6: Despite the detail in this section, I struggled to get a clear understanding of how Untrecht scale is traced to VCDT. A clear description of tank assignments and hierarchy is missing. I understand the path somehow went through the Tokyo Institute of Technology. But how did they assign values to the COS samples? The paragraph that starts “The δ34S values on the CVDT scale were assigned…” is vague on what tanks are being assigned through this process. If the assignments were done in Tokyo, why do they need to be reassigned in Utrecht? I suggest adding a table which lists the tanks, their COS concentrations, and how they were assigned, e.g. treated as primaries (from Tokyo) or secondaries (assigned in Utrecht).
- Eq. (3). I’m not sure I see the point of calculating sigma_total. Typically two types of error are relevant: How well can individual measurements be related to an internal lab scale? How well can the lab scale be related to scales in other labs? These two types of error require keeping sigma_cal distinct from the other two errors. Also, the quantity sigma_system was not clearly defined. Same as the “reproducibility” cited earlier?
- “Data corrections, quality check and long-term stability”. I was left without an understanding of the point at which the blank correction might have become problematic. Isn’t 5% already a bit large?
- “A target cylinder (5 L, dried ambient air filled in Greenland in 2017) was measured approximately weekly to monitor the long-term stability of the isotope measurements”. This sentence punctuates a break in the narrative thread. New paragraph, with a topic sentence?
- “The trapping efficiency was found to be 100 % for all injected volumes that were tested”. I’d have expected this to have uncertainty. May be expressed as greater than, as in > 99% or > 99.9%.
- “CO2 interference was specifically tested since it was suspected to be one of the larger peaks on the chromatogram and since its possible interference was also mentioned by Angert et al. (2019).” Wouldn’t this test have been sufficient to positively identify the CO _2_ peak on the chromatograph? If so, why did the earlier discussion suggest that this peak assignment was only tentative?
- Eq. (4) and (5). I’m unclear on why it is relevant to present functional fits to these data. Also, the data alone are not precise enough establish the exponential form. Why was this form chosen? Why not an inverse square root or other power law?
- Table 1. I don’t understand why the d34S values from Utrecht differ so much from the values in Japan. Does Utrecht keep a different dS34 standard? I presume this is related to the delta value relative to the instrument standard. But this is not set out clearly. It might help to create another symbol for this other scale, such as delta_internal. This is related to the point above about scale traceability.
- “we made sure always to measure a 3L zero air injection after a measurement of a gas ..” The blank correction sensibly depends only on what was measured before, not after a sample.

Is the rationale for developing the new method (or application) clearly explained?

Yes

Is the description of the method technically sound?

Yes

Are the conclusions about the method and its performance adequately supported by the findings presented in the article?

Yes

If any results are presented, are all the source data underlying the results available to ensure full reproducibility?

Partly

Are sufficient details provided to allow replication of the method development and its use by others?

Yes

Reviewer Expertise:

Measurements of CO2 and related atmospheric species.

I confirm that I have read this submission and believe that I have an appropriate level of expertise to confirm that it is of an acceptable scientific standard.

# Response

**Published:** 2022-03-02

**Authors:** Sophie Baartman

**Affiliations:** Utrecht University, The Netherlands

Thank you for the kind and very useful review. See below for our responses to the comments, questions and suggestions.

- *“Direct measurements of GPP are difficult to obtain, because flux measurements only yield the sum the two almost cancel-ling fluxes: GPP and respiration.” Needs to mention CO _2_ fluxes.*

This has now been added.

- *“The sulfur isotope ratios are reported as a δ values” Cut “a”.*

This has been changed.

- *“Newman et al. (1991) presented the first global estimate of 34S in tropospheric COS of 11 ‰..” – The absolute value of 11 ‰ is ambiguous without a discussion of the standard used in Eq. (2). Could be fixed by mention the standard earlier when presenting Eq. (2).*

The international standard VCDT is now mentioned already when introducing the δ value calculation and the full name mention of the VCDT has been removed from the method section.

- *“The destruction reaction of COS with hydrogen oxide radicals (OH)” - Aren’t these more normally called ­hydroxyl radicals?*

Indeed. This has been changed now.

- *“In short, the sample gas is first directed through a cooled Tenax trap..” I missed an explanation of why two cold traps at different temperature are needed. Why doesn’t the first trap achieve adequate focusing? Something to do with the materials in the trap and ability to warm quickly?*

An additional explanation is added later on in the paragraph where the pre-concentration system is described in more detail: “This second cold trap is used to collect and release all the COS at the same time, creating a narrower peak on the chromatograph than if only the first cold trap, which heats up slower, would be used.”

-  
*“After all the sample gas is injected” Context is not clear here. Injected where? What triggers stopping the sample flow?*

This sentence was indeed unclear. It is now changed into: “After the desired volume of sample gas is injected in the Tenax trap, the collected gas is released from the Tenax by heating the trap to 130 °C with a heating wire.”

- *Figure 2: I see four peaks on the left, not three, so the caption is confusing. Are we to ignore the first peak? For clarity, I suggest labeling the peaks: O _2_, CO _2_?, COS, organics?*

The smaller peak at the beginning of the chromatograph is indeed also O _2_ and is present because we constantly flush the IRMS source with O _2_ in between measurements. There is now an added sentence in the text explaining this: “Because the IRMS source is constantly flushed with O2 in between measurements, we also see half a peak of O2 at the beginning of the chromatograph.”. Some changes on figure 2 were already suggested by another reviewer. The inset is enlarged in the new version and labels for the peaks are now also added.

- *“Calibration” Section starting page 6: Despite the detail in this section, I struggled to get a clear understanding of how Untrecht scale is traced to VCDT. A clear description of tank assignments and hierarchy is missing. I understand the path somehow went through the Tokyo Institute of Technology. But how did they assign values to the COS samples? The paragraph that starts “The δ34S values on the CVDT scale were assigned…” is vague on what tanks are being assigned through this process. If the assignments were done in Tokyo, why do they need to be reassigned in Utrecht? I suggest adding a table which lists the tanks, their COS concentrations, and how they were assigned, e.g. treated as primaries (from Tokyo) or secondaries (assigned in Utrecht).*

The calibration process was indeed a bit complicated and therefore not easy to describe. Four calibration gases were produced in Japan, and their d34S values on the VCDT scale were calculated using the two methods described in the second paragraph of the calibration section (on-line and off-line method). Thus, these are our primary gases. These gases were then shipped to Utrecht and used to construct a calibration for the measurements on our IRMS. We have our own reference gas (the normal air cylinder from Greenland) which we calculated all our samples relatively to. Using the calibration gases from Japan, we constructed a calibration curve (figure 5) in which we plot the d33S_VCDT and d34S_VCDT against the d33S and d34S as measured in Utrecht (relatively to our own reference gas). This calibration curve was then used to assign a value on the VCDT scale to our reference gas, which is then used to calculate the VCDT values of measured samples. The synthetization and calibration procedure of the primary gases, performed at the Tokyo Institute for Technology is described in Hattori *et al*. (2015) and is therefore not described in this paper in much detail. We tried to reformulate the methods section on the calibration slightly so that it is a bit clearer. Table 1 contains all the isotope values from the measurements in Utrecht (calculated against our reference gas) and the ones performed in Japan on the VCDT scale. As we wanted to keep the results and methods separated, the table is in the calibration results section.

- *Eq. (3). I’m not sure I see the point of calculating sigma_total. Typically two types of error are relevant: How well can individual measurements be related to an internal lab scale? How well can the lab scale be related to scales in other labs? These two types of error require keeping sigma_cal distinct from the other two errors. Also, the quantity sigma_system was not clearly defined. Same as the “reproducibility” cited earlier?*

You are right. After some thinking from our side about our error propagation we indeed decided to make a difference between the error of our measurement system (how well our measurements can be related to our internal lab scale) and the calibration error, which represents how well our measurements can be related to the international VCDT scale. This has now been changed in the text and equation 3 has been adjusted to only include the reproducibility error and the nonlinearity error, which together make up our internal error. Because we are no longer incorporating the calibration error into the total reported error on individual measurements, the error for δ33S decreased slightly from 3.3‰ to 2.5‰ (see abstract). For δ34S it did not make a difference.

- *“Data corrections, quality check and long-term stability”. I was left without an understanding of the point at which the blank correction might have become problematic. Isn’t 5% already a bit large?*

The blank correction is not problematic. We use a blank correction to account for the “background” that is always in our system and therefore also in the sample measurements. This blank must be subtracted from the raw data in order to estimate the correct values of the samples. For this purpose, we need to determine the magnitude and stability of the blank.

- *“A target cylinder (5 L, dried ambient air filled in Greenland in 2017) was measured approximately weekly to monitor the long-term stability of the isotope measurements”. This sentence punctuates a break in the narrative thread. New paragraph, with a topic sentence?*

Yes indeed, good point. This has now been changed into a new paragraph and the first sentence has been altered slightly.

- *“The trapping efficiency was found to be 100 % for all injected volumes that were tested”. I’d have expected this to have uncertainty. May be expressed as greater than, as in > 99% or > 99.9%.*

This has now been changed into >99%, since indeed the uncertainty was not taken into account when mentioning the 100% trapping efficiency.

- *“CO2 interference was specifically tested since it was suspected to be one of the larger peaks on the chromatogram and since its possible interference was also mentioned by Angert et al. (2019).” Wouldn’t this test have been sufficient to positively identify the CO _2_ peak on the chromatograph? If so, why did the earlier discussion suggest that this peak assignment was only tentative?*

This section actually describes testing the possible effect of different substances being present in the gas mixture on the trapping efficiency of COS on the Tenax. Since Tenax also traps a small amount of CO2 on its surface, one might expect an interference on the trapping of COS if there are large amounts of CO2 present in a gas mixture. However, we did not see this interference and nor did Angert *et al. *(2019). This is a different interference than a possible overlapping of the peaks on the chromatograph, which we also checked.

- *Eq. (4) and (5). I’m unclear on why it is relevant to present functional fits to these data. Also, the data alone are not precise enough establish the exponential form. Why was this form chosen? Why not an inverse square root or other power law?*

The exponential function was chosen because this was the best fit for the data. We needed to have a continuous function in order to calculate the reproducibility for all the data. For the range of the ambient air measurement data of between 0.6 and 0.8 Vs, it does not matter that much which function was chosen. The estimate of the reproducibility will also improve as we will continue to gather more data.

- *Table 1. I don’t understand why the d34S values from Utrecht differ so much from the values in Japan. Does Utrecht keep a different dS34 standard? I presume this is related to the delta value relative to the instrument standard. But this is not set out clearly. It might help to create another symbol for this other scale, such as delta_internal. This is related to the point above about scale traceability.*

The d34S values of Japan are the values calibrated to the VCDT scale. The d34S values of Utrecht presented in this table are calculated relatively to our reference gas, which is the Greenland air cylinder. Thus, these d34S Utrecht values are not on the VCDT scale yet. We used the measurements in Japan to calibrate our measurements to the VCDT scale. We have this table in our paper to present the values used for the calibration line shown in figure 5. In figure 5 we use the d34S Utrecht and the d34S_VCDT off-line (done in Japan) to calibrate d34S. This could indeed be made clearer in this table, so we now changed d34S Utrecht into d34S_ref-Utrecht and d33S Utrecht into d33S_ref-Utrecht

- *“we made sure always to measure a 3L zero air injection after a measurement of a gas ..” The blank correction sensibly depends only on what was measured before, not after a sample.*

This is true. This sentence was not clear. What we meant was that we measured a zero air gas after a high mixing ratio sample in order to minimize the memory effect on the next sample measurement. This sentence has now been changed to make this clear: “However, we made sure to always measure a 3 L zero air injection after a measurement of a gas with higher COS mixing ratio, in order to minimize a potential memory effect on the subsequent measurement.”

# Reviewer report: Reviewer response for version 1

**DOI:** 10.21956/openreseurope.14959.r27642

**Published:** 2021-10-15

**Authors:** Sinikka Lennartz

**Affiliations:** 1 ICBM-MPI Bridging Group for Marine Geochemistry, Institute for Chemistry and Biology of the Marine Environment, University of Oldenburg, Oldenburg, Germany

The article by Baartman *et al*. describes a new method to measure the isotope ratios of sulphur in the trace gas carbonyl sulphide (COS) in ambient air samples. The motivation is laid our clearly, and the improvement of being able to measure low sample volumes reliably is an important one. The authors describe in detail the set-up and the results of various tests they performed to ensure the stability of the system and the absence of contamination. The latter is crucial for any system measuring COS, as many materials have been identified as a source of contamination previously. In addition to the description of the measurement system, the authors also apply the new technique to ambient air samples of different origin (ambient air at Utrecht, tunnel system) and report new COS isotope data that is of scientific relevance. I especially acknowledge the great level of detail given to describing the performed tests, the system and the preparation of standard gases. The paper is clearly structured and well written. I recommend this article for indexing, after some minor comments have been addressed, which I include in the following.

 Abstract:

- The causality between 620 ppt (i.e. elevated COS mixing ratio) and the origin (i.e. not from anthropogenic emissions) was not clear to me – in the main text, you support the origin mainly by the high isotopic ratio, not by the high concentration. I suggest to rewrite the sentence.
- Strictly speaking, “40 ppt” is not a trend, as a trend should relate to a time unit. Can you say 40 ppt over 3 months or sth, to make it more clear?

Introduction:

- First paragraph: COS is the main source to stratospheric aerosols in volcanically quiescent periods only. I would suggest rewriting as “a major source” or “the main source in the absence of volcanic eruptions”.
- Paragraph 2: Consider citing some of the work by Protoschill-Krebs 1992/1996 to give credit to their work on how COS is taken up by plants, in addition to the review by Whelan et al., 2018.

Methods:

- Fig. 2: I would suggest changing the size of the two panels: the close-up on the OCS peaks seems to me the most interesting one, and the one with the other peaks could be the smaller one.
- The sentence “The presence of scale contraction was checked by looking at the slope of the calibration line” and the following one could be revised to “A slope of the calibration line >1 was considered as an evidence of scale contraction, and a correction method was developed.”
- Reference COSANOVA, n.d.: I would suggest putting the link directly in the text.
- In the section on Data corrections: You state some data that are clearly outliers were not included into the final dataset. Can you roughly give an estimate how often such outlier occur?
- When you describe the “Additional tests” in the method sections, you already give some results there. I would suggest moving that to the results section, to let the reader find the information he or she looks for where it is expected.
- Highway tunnel measurements: You already state in the text that the CO2 concentration increases throughout the tunnel due to accumulation, but the canister for COS is mainly filled at the beginning. Assuming COS and CO2 concentrations are correlated in the tunnel (which is safe to assume if both have traffic emissions as their main source), what does that mean for the COS:CO2 ratio that you give? Is the resulting ratio rather a lower limit for the COS:CO2 ratio?

Results

- Fig. 3: the curve for d33S seems to strongly depend on the point at 0.4 Vs. Any idea on what causes this outlier, which is also a “low outlier” for d34S? Can you say sth on how consistent this pattern with this one outlier at 0.4 Vs is? How different is the fitted curve if the one at 0.4 Vs is omitted?
- Fig 3 caption/text: Method and caption say n=7-8, but text says n=6-8, please correct appropriately.
- To show the reproducibility (precision), I would have expected to see a figure with all individual data points. In the method section you state that reproducibility is tested by having 1L, 2L and 3L in 7-8 repetitions for two gases. I would suggest showing this in detail.
- Nonlinearity: You state that the nonlinearity is small and that only a small correction was needed. I may be missing sth here, but which correction did you actually apply? Can you give the exact equation?
- Can you describe why the scatter is so different for d34S and d33S in Fig. 4 and especially Fig. 5?
- Fig 6: It is good to be transparent about the second canister as a replacement after the first one was exhausted, and you state that clearly in the methods. For increasing the visibility of the plot, I suggest writing in the caption that both target gases are designed to be comparable, i.e. should have the same concentration. At least to me, this was confusing at the first look because they have different colors, so I had to go back to the method section to check what the difference between the two gases is.
- Measurements in Utrecht: “More generally, European anthropogenic COS emissions in the Netherlands are likely small” – I think I understand what you mean, but the sentence needs revising. Maybe: “More generally, the impact of European anthropogenic COS emissions in the Netherlands are likely small”. Still, how do you explain the increase in mixing ratio of COS that seemed to decrease again when CO2 and CH4 mixing ratios were still high?
- Conclusion: could you compare the errors to the errors of the previous methods? What is the final take on the trade-off between sample volume and uncertainty compared to the other methods?

I confirm that the links for underlying and extended data are working, and the data is fully accessible. I randomly checked some of the files and realised that e.g. in COS_isotopes_ambient_air_data_Baartman_et_al.xlsx, the units are missing (while they are present in other files). It becomes clear from the context that it must be ppt, but would be helpful to add units for non COS-experts.

Is the rationale for developing the new method (or application) clearly explained?

Yes

Is the description of the method technically sound?

Yes

Are the conclusions about the method and its performance adequately supported by the findings presented in the article?

Yes

If any results are presented, are all the source data underlying the results available to ensure full reproducibility?

Yes

Are sufficient details provided to allow replication of the method development and its use by others?

Partly

Reviewer Expertise:

Marine carbonyl sulfide cycling, measurement of carbonyl sulfide via integrated cavity output spectroscopy, global budget of carbonyl sulfide

I confirm that I have read this submission and believe that I have an appropriate level of expertise to confirm that it is of an acceptable scientific standard.

# Response

**Published:** 2022-03-02

**Authors:** Sophie Baartman

**Affiliations:** Utrecht University, The Netherlands

Thank you for the very kind review and the useful comments, suggestions, and questions. See below for our responses.

Abstract

- *The causality between 620 ppt (i.e. elevated COS mixing ratio) and the origin (i.e. not from anthropogenic emissions) was not clear to me – in the main text, you support the origin mainly by the high isotopic ratio, not by the high concentration. I suggest to rewrite the sentence.*

The sentence is now rewritten to include the relatively high isotope ratio.

- *Strictly speaking, “40 ppt” is not a trend, as a trend should relate to a time unit. Can you say 40 ppt over 3 months or sth, to make it more clear?*

The sentence is changed.

Introduction

- *First paragraph: COS is the main source to stratospheric aerosols in volcanically quiescent periods only. I would suggest rewriting as “a major source” or “the main source in the absence of volcanic eruptions”.*

Good point. It is now added to the sentence.

- *Paragraph 2: Consider citing some of the work by Protoschill-Krebs 1992/1996 to give credit to their work on how COS is taken up by plants, in addition to the review by Whelan et al., 2018.*

Both papers are added to the citation and to the reference list.

Methods

- *Fig. 2: I would suggest changing the size of the two panels: the close-up on the OCS peaks seems to me the most interesting one, and the one with the other peaks could be the smaller one.*

The figure is now changed. The one with all the peaks is still the larger one because I wanted to also be able to show the entire chromatograph well, with the square peaks. But now the inset is much larger and the thickness of the lines and the font there are also changed to make it all more visible and readable.

- *The sentence “The presence of scale contraction was checked by looking at the slope of the calibration line” and the following one could be revised to “A slope of the calibration line >1 was considered as an evidence of scale contraction, and a correction method was developed.”*

Good suggestion. The sentence is changed into: “A slope of the calibration line >1 was considered as an evidence of scale contraction and, if needed, a correction method was developed.”

- *Reference COSANOVA, n.d.: I would suggest putting the link directly in the text.* 

I suggested to the editors to include the link to the website directly in the text instead of in the reference list.

- *In the section on Data corrections: You state some data that are clearly outliers were not included into the final dataset. Can you roughly give an estimate how often such outlier occur?*

I checked the last 300 measurements (including zero air and reference gas measurements), of which 7% were flagged due to the various reasons mentioned in the paper (error in pre-concentration, outlier due to other reasons). A sentence was included in this section that mentions the occurrence of outliers that were flagged.

- *When you describe the “Additional tests” in the method sections, you already give some results there. I would suggest moving that to the results section, to let the reader find the information he or she looks for where it is expected.*

The results of this section used to be in an appendix to the paper as we thought they did not necessarily have to be included in the main results of the paper. From the editor, we received the suggestion to include these results already in the methods section. We think this is more convenient than adding another paragraph to the results section. Also when one wants to reproduce the methods they can immediately find the outcomes of the tests we performed without having to look them up in the results section.

- *Highway tunnel measurements: You already state in the text that the CO2 concentration increases throughout the tunnel due to accumulation, but the canister for COS is mainly filled at the beginning. Assuming COS and CO2 concentrations are correlated in the tunnel (which is safe to assume if both have traffic emissions as their main source), what does that mean for the COS:CO2 ratio that you give? Is the resulting ratio rather a lower limit for the COS:CO2 ratio?*

The reported COS:CO2 ratios are enhancements above the background mixing ratios of COS and CO2. Thus, the fact that most sampling took place closer to the entrance of the tunnel will not affect the ratios as we compensate for the amount of background COS and CO2 that are also present in the samples. The second paragraph of the highway tunnel measurements results section states: “From these values we calculated the COS/CO2 **enhancement** ratio…”

Results

- *Fig. 3: the curve for d33S seems to strongly depend on the point at 0.4 Vs. Any idea on what causes this outlier, which is also a “low outlier” for d34S? Can you say sth on how consistent this pattern with this one outlier at 0.4 Vs is? How different is the fitted curve if the one at 0.4 Vs is omitted?*

The precision of the mass spectrometer can change slightly sometimes due to several reasons (change of filament, focus settings among others). We performed a test at 0.4 Vs and at that moment the precision was very good. It is unclear as to why exactly the precision was that good at that moment. If we omit this outlier, we get this figure linked here: *https://s3-eu-west-1.amazonaws.com/openreseurope/linked/194968.figure3_no_0.4Vs.pdf*

 We can see that the regression line indeed changes quite a bit towards the lower peak areas, but hardly at the higher peak areas of >0.5 Vs. As all the results presented in this paper have a peak area of >0.5 Vs, and most even >0.6 Vs, changing this regression line for calculating the errors will not make a significant difference for the measurement errors. However, for future measurements, we will do more measurements to characterize the reproducibility (system precision). 

- *Fig 3 caption/text: Method and caption say n=7-8, but text says n=6-8, please correct appropriately.*

It indeed should have been n=6-8. It is now changed in the methods section and in the figure 3 caption.

- *To show the reproducibility (precision), I would have expected to see a figure with all individual data points. In the method section you state that reproducibility is tested by having 1L, 2L and 3L in 7-8 repetitions for two gases. I would suggest showing this in detail.*

I think a figure with all the measurements will be a bit less informative than figure 3. Since we used different gases to determine the precision and we also calculate the precision using this student t-distribution. If you show all the measurements in one plot this will be a big cloud of points with different colors (for the different gases) and it will not become immediately apparent what the precision is, depending on the peak area (sample volume).

- *Nonlinearity: You state that the nonlinearity is small and that only a small correction was needed. I may be missing sth here, but which correction did you actually apply? Can you give the exact equation? *

The equations are now included in the section (equation 6 and 7). I also noticed that the equation numbering in version 1 of the paper was incorrect. This has been solved now.

- *Can you describe why the scatter is so different for d34S and d33S in Fig. 4 and especially Fig. 5?*

Our measurement precision (reproducibility) is lower for d33S than for d34S. This is due to the lower natural abundance of ^33^S (0.75%) compared to ^34^S (4.25%). From this, one would expect to have approximately a 2 times higher reproducibility for d34S than for d33S. We have slightly lower precision for d33S than d34S (2.1‰ for d33S and 0.4‰ for d34S). That is why we see so much more scatter for d33S compared to d34S in the data.

- *Fig 6: It is good to be transparent about the second canister as a replacement after the first one was exhausted, and you state that clearly in the methods. For increasing the visibility of the plot, I suggest writing in the caption that both target gases are designed to be comparable, i.e. should have the same concentration. At least to me, this was confusing at the first look because they have different colors, so I had to go back to the method section to check what the difference between the two gases is.*

Good point. An extra explanation is added to the caption of figure 6. However, they were not really designed to have the same concentration. As can be seen in figure 6, the second target gas does have higher mixing ratio than the first one. They were simply gases that were stable in larger cylinders so that they would not be exhausted that quickly.

- *Measurements in Utrecht: “More generally, European anthropogenic COS emissions in the Netherlands are likely small” – I think I understand what you mean, but the sentence needs revising. Maybe: “More generally, the impact of European anthropogenic COS emissions in the Netherlands are likely small”. Still, how do you explain the increase in mixing ratio of COS that seemed to decrease again when CO2 and CH4 mixing ratios were still high?*

This sentence was indeed not clear. It is now changed into: “More generally, the impact of European anthropogenic emissions on the COS budget in the Netherlands is likely small, because we generally do not see events with high COS mixing ratios and/or very depleted values of δ 34S during our measurement period.” About the measurements in figure 8: we do indeed not completely understand why the COS mixing ratio increased during the pollution event and then decreased again while CH4 and CO2 kept increasing. We expected the COS to increase together with CH4 and CO2. However, as these are measurements taken with approximately 2-hour increments, we can expect some variability in the air trajectories between measurements. It could be that this one high COS measurement picked up a local source of COS that the other measurements did not see. CH4 and CO2 sources are more widespread (such as traffic and industries). However, COS sources are likely more like individual point sources which can either be picked up by the measurement or not, if the trajectory changes slightly. Furthermore, during these measurements, we had very stable cold weather in Utrecht, which was likely trapping pollution during the night. The 8 ^th^ measurement (with high COS mixing ratio) was taken around 9:30 in the morning on the 2 ^nd^ of December. Afterwards, there were some reference gas measurements (hence the time gap in figure 8) and the next ambient air measurement was taken at around 13:30. In the afternoon, boundary layer mixing will have occurred, so we probably also see a reflection of these different conditions.

- *Conclusion: could you compare the errors to the errors of the previous methods? What is the final take on the trade-off between sample volume and uncertainty compared to the other methods?*

Angert *et al.* (2019) report a precision of ≤ 0.5 ‰ for ambient air measurements taken from samples in 2.25 L canisters with 9 repetitions. They use an ICP-MS which is a very different and much more expensive type of mass spectrometer, so our measurements are difficult to compare to theirs in terms of precision and trade-offs. Kamezaki *et al*. (2019) report a precision of 0.4 ‰ for measurements where COS is pre-concentrated from 500 L of air and then measured using GC-IRMS. Our method also uses GC-IRMS but we correct for the nonlinearity and then are able to measure with 0.9 ‰ precision for a 4 L sample, which are much easier to collect in the field or in experiments than 500 L of air. Because our method uses less air we possibly have fewer errors during the pre-concentration phase. And also because of this small sample size, we are able to use an air cylinder with ambient COS mixing ratio as a reference gas. Thus, the reference measurements and sample measurements proceed in the same way. The downside of our method is that we have to keep characterizing the nonlinearity of the measurements as this behavior could change over time. These testing measurements are somewhat time-consuming and would not be necessary this often if one would measure outside of the nonlinear regime (e.g. much larger sample volumes). We do have about double the standard deviation of the measurements by Kamezaki *et al.* (2019), however, our sample standard deviation is usually still below 1 ‰ for d34S which is good enough to see differences in ambient COS isotopic composition. Furthermore, we do not have an independent calibration method, thus the accuracy of our results depends on the accuracy of the calibration measurements in Japan and on the stability of the calibration gases provided to us. I confirm that the links for underlying and extended data are working, and the data is fully accessible. I randomly checked some of the files and realised that e.g. in COS_isotopes_ambient_air_data_Baartman_et_al.xlsx, the units are missing (while they are present in other files). It becomes clear from the context that it must be ppt, but would be helpful to add units for non COS-experts. Thank you for pointing this out. I actually found that more files were missing units. I included all the units now and the new files can be found in the extended data with the new DOI: https://doi.org/10.5281/zenodo.6319758
